# Supervised latent factor modeling isolates cell-type-specific transcriptomic modules that underlie Alzheimer’s disease progression

**DOI:** 10.1038/s42003-024-06273-8

**Published:** 2024-05-17

**Authors:** Liam Hodgson, Yue Li, Yasser Iturria-Medina, Jo Anne Stratton, Guy Wolf, Smita Krishnaswamy, David A. Bennett, Danilo Bzdok

**Affiliations:** 1 School of Computer Science, McGill University, Montréal, QC Canada; 2 Mila - Quebec Artificial Intelligence Institute, Montréal, QC Canada; 3 McConnell Brain Imaging Centre (BIC), MNI, Faculty of Medicine, McGill University, Montréal, Canada; 4 Neurology and Neurosurgery Department, Montreal Neurological Institute (MNI), Faculty of Medicine, McGill University, Montréal, Canada; 5 Ludmer Centre for Neuroinformatics and Mental Health, McGill University, Montréal, Canada; 6 Department of Mathematics & Statistics, Université de Montréal, Montréal, Canada; 7 Department of Computer Science, Department of Genetics, Yale University, New Haven, CT USA; 8 Rush Alzheimer’s Disease Center, Rush University Medical Center, Chicago, IL USA; 9 Department of Biomedical Engineering, Faculty of Medicine, McGill University, Montréal, QC Canada; 10 The Neuro - Montréal Neurological Institute, McConnell Brain Imaging Centre, Faculty of Medicine, McGill University, Montréal, QC Canada

**Keywords:** Computational models, Gene expression

## Abstract

Late onset Alzheimer’s disease (AD) is a progressive neurodegenerative disease, with brain changes beginning years before symptoms surface. AD is characterized by neuronal loss, the classic feature of the disease that underlies brain atrophy. However, GWAS reports and recent single-nucleus RNA sequencing (snRNA-seq) efforts have highlighted that glial cells, particularly microglia, claim a central role in AD pathophysiology. Here, we tailor pattern-learning algorithms to explore distinct gene programs by integrating the entire transcriptome, yielding distributed AD-predictive modules within the brain’s major cell-types. We show that these learned modules are biologically meaningful through the identification of new and relevant enriched signaling cascades. The predictive nature of our modules, especially in microglia, allows us to infer each subject’s progression along a disease pseudo-trajectory, confirmed by post-mortem pathological brain tissue markers. Additionally, we quantify the interplay between pairs of cell-type modules in the AD brain, and localized known AD risk genes to enriched module gene programs. Our collective findings advocate for a transition from cell-type-specificity to gene modules specificity to unlock the potential of unique gene programs, recasting the roles of recently reported genome-wide AD risk loci.

## Abstract (web summary)

Designing a supervised latent factor framework for snRNA-seq human brain, the authors find distinct Alzheimer’s-predictive gene modules across celltypes, suggesting subcelltype disease progression trajectories.

## Introduction

Late-onset Alzheimer’s disease (AD) is a neurodegenerative disorder with a large burden on the global healthcare system that is being exacerbated by our aging societies. The gradual progression of AD begins with mild cognitive impairment and cumulates in severe memory loss and death, often many years or decades after onset^1^. While identified >100 years ago^2^, the underlying disease pathways of AD remain obscure. Increasingly large genome-wide association studies (GWAS) have identified genetic variants that contribute to AD risk, with the largest study to date identifying 38 risk loci at genome-wide significance^3^. In parallel, bulk transcriptomic measurements have enabled functional views on the genetic mechanisms of disease onset and progression from a tissue-average perspective^4^. More recently, high-throughput single-nucleus RNA sequencing (snRNA-seq) of post-mortem human brain tissue has enabled the identification of gene expression effects at the resolution of brain cell-types. This finer resolution has made it apparent that different cell-types play different roles in the pathogenesis of neurodegenerative diseases. For example, at the cellular resolution, *APOE*, the top AD risk gene, was found to be simultaneously upregulated in microglia but downregulated in astrocytes in AD patients^5^—an important insight previously inaccessible to methods unable to resolve gene expression profiles in individual cells. Additionally, snRNA-seq studies have demonstrated that previous bulk RNA-seq studies were dominated by RNA originating from abundant neurons and oligodendrocytes, potentially eclipsing the signal from cell-types less abundant in the brain such as microglia^5^. Many single-nucleus studies continue to underscore the importance of glial cells—and particularly microglia due to their high concentration of expressed GWAS risk loci—as playing a major role in AD pathophysiology^6–8^.

Despite the growing number of genomic studies of AD in the brain from a single-cell perspective, it remains challenging to understand which particular biological processes and specific molecular pathways are implicated in AD pathophysiology. One common approach to analyzing snRNA-seq cell transcriptomes leans heavily on quantifying differential expression between persons with and without AD, one gene at a time. While this single-gene approach has uncovered insights about AD pathophysiology, isolated case-control deviations in a particular gene often evade direct interpretation. Similarly, risk loci identified at the level of human populations through GWAS are often challenging to resolve in the context of individual cell-types and molecular processes. As highlighted by co-expression network approaches^9^, it is probably collections of pathologically expressed genes that together act in concert to drive the disease phenotype. We believe that identifying and characterizing such disease-driving groups is essential for ultimately identifying the causes of AD.

Although individual genes may not be detected as being significant in differential expression, if considered in the full picture of companion genes, we may reveal their role in broader biological systems^10^. Machine learning solutions are naturally suited to consider the totality of tens of thousands of expressed genes simultaneously^11^. Therefore, this research paradigm may better accommodate the functional interplay expected between concomitantly transcribed “cliques” of genes in the full functional context of a cell. Unsupervised latent factor models, such as PCA, t-SNE, and UMAP, are commonly used in snRNA-seq processing pipelines, typically for the preprocessing and visualization of collections of high-dimensional transcriptomes. However, this un-guided latent structure discovery is blind to valuable contextual information such as the disease status of the cells and other external clinicopathological markers. Therefore, the geometry of the derived embedding space dimensions may be quite different after accounting for the external information related to the disease under study. Because of this reliance on un-guided structure discovery with incomplete contextual information, previously discovered latent spaces for cell transcriptomes may have provided only partial insights into the biological phenomena underlying the disease state. A similar insensitivity to supervising information applies to deep learning variants of unsupervised latent factor models, such as variational autoencoders^12^. While some variants do anchor the non-linear embedding to biological pathway information to enhance interpretability^13,14^, this latent space is still not extracted by conditioning on a provided disease phenotype. Machine learning models whose estimation takes into account known disease status, but lack latent factor extraction, have been applied to snRNA-seq data^15^. For example, the use of diffusion-condensation combined with supervised graph signal processing can help zoom in on the appropriate level of detail to find relevant subpopulations^16^. However, these supervised methods do not simultaneously perform latent structure discovery. Hence, these modeling approaches lack the interpretability that comes from learning a compressed representation of several sources of gene activity variation. Our study attempts to remedy this shortcoming by encouraging the discovery of intrinsic structure within gene expression patterns observed in a specific cell-type, explicitly guided by a target phenotype of scientific interest: AD diagnosis.

Here, we implement a supervised latent factor framework from machine learning tailored to improve the interpretability of snRNA-seq transcriptome effects at the granularity of distinct gene expression programs in AD. We systematically zoomed in on each of the six major cell-types (excitatory and inhibitory neurons, oligodendrocytes and their precursors, astrocytes, and microglia) of the human prefrontal cortex (BA10). The analyzed dataset provided ~70,000 cells sampled from 48 age- and sex-matched donors from the Religious Orders Study or Rush Memory and Aging Project (ROSMAP) cohort^5,17^. We could thus demonstrate that our supervised latent-factor framework distinguishes healthy cells from diseased cells by learning biologically interpretable modules within each cell-type, and that these modules could be linked to biologically meaningful gene programs from large, curated ontologies. We first fit disease-discriminative models to the gene expression data to identify cell-type-specific modules. Leveraging established annotations from gene program databases such as Gene Ontology, we applied gene set enrichment analysis (GSEA) to the gene importance scores for each latent module. We show that these learned modules identify distinct biological processes and pathways that are predictive of AD, going beyond single genes found using differentially expressed genes. We then investigate each gene module to understand its connection to the most recent set of 38 AD genome-wide significant risk genes across cell-types and their modules. Finally, we explore the possible interactions between modules found in different cell-types, quantifying the level of cell-cell interaction present in AD. Our results underscore the value of dedicated machine learning tools that consider the expression of all genes simultaneously to isolate several distinct latent gene expression modules, within a given cell-type, that are predictive of AD.

## Results

### Supervised latent-factor modeling identifies cell-type-specific gene modules implicated in AD

We hypothesized that brain cells sampled from persons with AD could be differentiated from cells from persons without AD based on a subset of underlying gene expression groups—here termed modules. We used partial least squares discriminant analysis (PLS-DA), a class of supervised latent-factor model, to distinguish between these two phenotypes while simultaneously uncovering interpretable latent modules. PLS-DA was a natural choice of method because it is a model that exploits the principle of parsimony by learning to separate target classes based on a number of underlying groups of expressed genes. These derived modules each prioritize different groups of genes that explain cell-type-specific differences between persons with and without AD^18^.

**Fig. 1:**
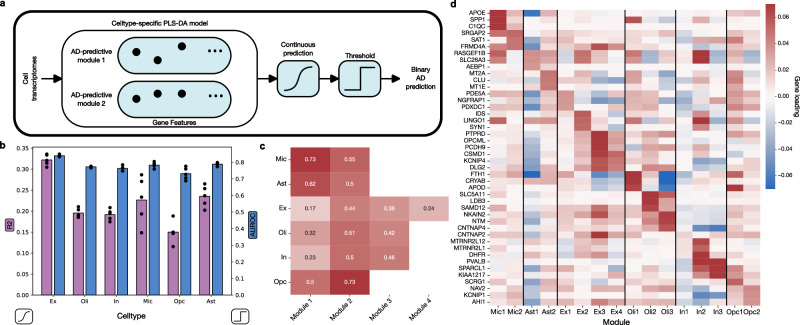
Latent-factor modeling reveals gene expression modules predictive of AD pathology in each brain cell-type. **a** We devised a pattern-learning approach for AD classification (partial least squares discriminant analysis, PLS-DA), and trained a dedicated model for each cell-type to distinguish between cells originating from brain tissue of persons with and without AD. Each cell-type-specific model was estimated on the gene expression profiles from all tissue donors, and learned a number of discriminative latent transcriptomic modules. Each of these module shows the predictive role of each gene in a collective subspace that maximizes separation according to disease status. To avoid overfitting, the number of modules for each cell-type was selected based on five-fold cross-validation, using the out-of-sample area under the ROC curve (AUROC) as the selection criterion. **b** We found that our model was able to successfully distinguish between the no AD and AD classes in all cell-types (*n* = 48 donors). AUROC in unseen cell transcriptomes ranged from 0.731 (s.d. 0.033) across cross-validation folds for OPCs up to 0.839 (s.d. 0.006) for excitatory neurons, and coefficient of determination (*R*^2^) ranged from 0.150 (s.d. 0.023) for OPCs up to 0.328 (s.d. 0.017) for excitatory neurons. **c** We calculated the association strength between the projection of the cells onto their corresponding latent modules and the binary diagnosis vector. We found that the first microglia and second OPC modules have the highest links to AD, whereas the first excitatory and inhibitory modules had the weakest AD links. The number of selected modules ranged from two (microglia, astrocytes, OPCs) to four (excitatory neurons). **d** Each gene expression module encapsulates a unique set of roles assigned to the candidate genes, where the positive weights flag higher transcript level to be indicative of AD cells (+), whereas negative expression weights are indicative of healthy cell samples (−). We visualized the weights corresponding to the top three genes in each module. We found several known AD GWAS risk gene among these top genes, including *APOE* and *CLU*. This provides strong evidence that each module captures a distinct set of genes, both relative to other modules for the same cell-types and across cell-types.

We analyzed an snRNA-seq dataset containing gene expression profiles from 70,634 cells, collected from the prefrontal cortex of 48 subjects (age/sex-balanced with 24 persons with and 24 without AD) from the ROSMAP study on aging and dementia^5^. In each of the six previously identified cell-types (excitatory/inhibitory neurons, oligodendrocytes, oligodendrocyte precursor cells (OPCs), microglia, and astrocytes), we trained our PLS-DA model to distinguish between cells originating from persons with or without AD, based on the normalized expression of 17,926 protein-coding genes (Fig. 1a). That is, each separate classifier model was trained on all cells of a given cell-type originating from all 48 subjects. By analyzing each cell-type independently, we aimed to find cell-type-specific effects, as opposed to effects common to all cell-types affected by AD. The disease classification models for each brain cell-type extracted several latent modules. Each cell-type module defined a collection of gene effects in that cell-type that collectively identified expression signatures that distinguished AD individuals’ cell transcriptomes.

Since it is not known a priori how many modules should be expected in each cell-type, we treated the number of modules as a model hyperparameter to be determined in a data-driven fashion. Independently in each cell-type, we trained the classification model using a principled nested 5-fold cross-validation framework: the captured cell transcriptomes were randomly divided into five data splits, then the model was fitted on four of the data splits (training set) and evaluated on the remaining unseen transcriptomes (validation set). This was repeated for the remaining groups, resulting in five estimates of how the model is expected to distinguish between unseen AD vs control transcriptomes of the same cell-type. This scheme identified an optimized number of modules in each cell-type that resulted in the maximum classification performance on unseen brain cells. This minimizes the possibility of overfitting to idiosyncrasies in the data. The optimal number of modules varied from two for OPCs and microglia, to four for excitatory neurons.

Our AD classification model showed robust discrimination performance on unseen brain cell transcriptomes in all examined cell-types, as measured by the area under the receiver operating characteristic curve (AUROC) (Fig. 1b). The mean and standard deviation of the AUROC scores obtained from the nested 5-fold cross-validation provided an overall assessment of the PLS-DA model’s ability to predict a cell’s AD phenotype. All cell-types were successfully discriminated above 0.5 random chance. Based on all the extracted modules, the highest AUROC was obtained for excitatory neurons, at 0.839 (s.d. 0.006), while the lowest was 0.731 (s.d. 0.033) for OPCs. Keeping only the most individually discriminative module for each cell-type, microglia showed the highest prediction performance with an AUROC of 0.724 (s.d. 0.030), compared to 0.589 (s.d. 0.009) for excitatory neurons, the cell-type with the highest overall classification performance. This observation suggests that the disease-predictive signal is concentrated in a single module of expressed genes in microglia, whereas it is distributed over multiple modules in excitatory neurons (Fig. 1c). We further show that the genes that are the top contributors to the predictive performance are distinct for each module (Fig. 1d).

### Cell-type-specific modules capture AD-related gene expression programs

To ground our identified cell-type modules in known biological processes and molecular pathways, we performed gene set enrichment analysis (GSEA) using the gene combinations corresponding to each identified transcriptomic module. This analysis allowed us to identify enriched gene sets, or programs, from large, annotated collections: Gene Ontology (Biological Processes), WikiPathways, and Panther Pathways. GSEA^10^ takes as input an ordered list of genes and looks for a statistically significant overabundance of genes from annotated gene sets at the top/bottom of this list. In this analysis we used the PLS-DA module weights to rank the genes in each module, as they represented the importance of that individual gene in that module in predicting the cell’s classification. Therefore, finding a concentration of genes of interest in terms of highest ranked genes represented a meaningful signal corresponding to that gene program.

**Fig. 2:**
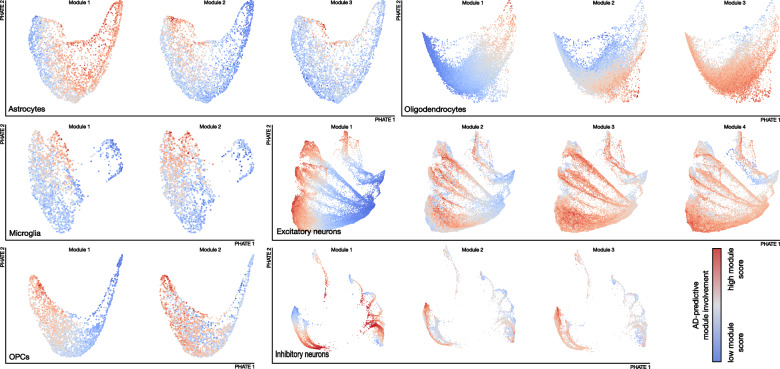
Different gene modules isolate distinct AD-related processes in each brain cell-type. We visualize the transcriptomes of all cells from each of the six brain cell populations in two dimensions using PHATE. Each cell (dot) in this visualization is then colored to indicate the relative strength (‘module score’) of a given functional gene module pattern in that cell’s transcriptome. This module involvement (i.e., PLS-DA projection of a cell’s transcriptomes) represents how strongly a given cell’s gene expression is aligned with the AD-predictive gene signatures identified in that module (red=higher expression, blue=lower expression). Supplementary Fig. 2 shows the same PHATE visualizations colored by binary donor diagnosis. While the PHATE visualization is not itself used to perform any analysis, it does help emphasize how often distinct subgroups of cells are identified by the different modules: different learned modules for each cell-type generally flag different groups of AD cells within each cell-type. For each module, we then performed gene set enrichment analysis (GSEA) and identified robust enrichment of biological processes through contextualization by means of Gene Ontology (Biological Processes), Wikipathways, and Panther Pathways, based on normalized enrichment score (NES). By inspecting the resulting lists of enriched gene sets, we were able to identify the dominant predictive set of biological signaling cascades in each module for each cell-type. We show that our gene modules can be localized to distinct subspaces in the spanned whole-transcriptome representation, many of which are indicative of well-established biological pathways that are associated with AD, while others point to new gene programs worthy of further investigation.

Here we summarize the main themes of the cell-type-specific predictive modules, which are visualized in Fig. 2.

In microglia, the leading disease-predictive module was enriched for gene programs related to the activation of microglial cells in response to an external stimulus, phagocytosis, and response to amyloid-beta plaque. Specifically, this gene expression module’s prioritized genes were found enriched in immune-activation-related programs including “microglial cell activation”, “regulation of complement activation”, and “*TYROBP* causal network in microglia”. Indeed, microglia under homeostatic conditions are known to be constantly probing their environment^19^, and can be activated by a variety of immune receptors^20^, which leads to phagocytosis and the release of signaling molecules to recruit other immune cells. Additionally, the complement system is involved in the innate immune response and can mediate phagocytosis^21^, and it was previously reported to be involved in AD^22^. Complement receptor *CR1* is a known GWAS risk locus for AD^23^. The *TYROBP* causal network was initially identified in mice, where *TYROBP* was pinpointed as the key regulator of a microglia immune module associated with AD pathophysiology^9^. In addition to gene programs related specifically to microglia activation, we also found relevant enrichments related to a general immune response, including “positive regulation of inflammatory response”, “positive regulation of tumor necrosis factor superfamily cytokine production”, and “regulation of interleukin-6 production”. Additionally, we found enrichments related to phagocytosis, including “microglia pathogen phagocytosis pathway”. One of the recognized roles of microglial is to engulf and clear debris, including apoptotic neurons^24^ and amyloid-beta^25^, a process which also involves the AD risk locus *TREM2*, discussed later. Finally, gene programs related to amyloid-beta included “regulation of amyloid-beta formation”. Activated microglia are known to congregate around amyloid-beta plaques, forming a protective barrier around these deposits^26^.

The second most important gene expression module in microglia was functionally related to the activation of the MAPK/ERK signaling pathway by cell surface receptor stimulation, and the toll-like receptor cascade. Enrichments for the MAPK/ERK signaling pathway included “EGF/EGFR signaling pathway”, “MAPK Signaling Pathway”, and “Ras signaling”. The MAPK pathway is critical to the increase in pro-inflammatory cytokines produced by microglia under stress. For example, this pathway can be triggered by amyloid-beta or toll-like receptors^27^, which are also implicated in this module. These pathways have previously been identified as playing a role in the inflammatory response present in AD. Specifically, it was found that ERK phosphorylation was a regulator of microglial pro-inflammatory immune response in mouse models of AD^28^. Gene programs related to the toll-like receptor cascade included “toll-like receptor signaling pathway”. It has indeed been suggested that targeting the TLR4, which triggers the MAPK pathway in microglia, could be a therapeutic target for AD^29^.

Hence, while microglia activation has been identified as one of the hallmarks of AD, our approach disentangled two unique modules that differentiate microglia originating from the brains of persons with and without AD pathology. Our findings underline the MAPK/ERK signaling pathway as being strongly predictive of positive disease status, in addition to the classical microglial immune activation pathways.

In astrocytes, the leading AD-predictive module related specifically to biological programs revolving around extracellular structure organization. Their enrichments included “extracellular matrix organization”, “cell junction assembly”, and “regulation of cell-cell adhesion”. The extracellular matrix includes the basement membrane, which helps maintain the blood-brain barrier integrity by connecting astrocytic endfeet to endothelial cells^30^. Blood-brain barrier dysfunction is strongly suspected to be implicated early in AD and other neurodegenerative disorders^31^. Furthermore, APOE4, the top risk variant associated with the development of late onset AD, is known to result in a thinning of the vasculature of the basement membrane and a breakdown of the blood-brain barrier in AD^32^. Astrocytes can influence the endothelial tight junctions which seal the BBB^33^, as well as form their own tight junctions. The loss of endothelial tight junctions is common in AD progression and is correlated with synaptic degradation^34^.

The second module in astrocytes was centered on gene programs related to peptide biosynthesis, neurogenesis, and the response to ion homeostasis, such as that of copper. The gene programs related to peptide synthesis included “peptide biosynthetic process”, and astrocytes are known to secrete the amyloid-beta peptide^35^, one of the primary biomarkers in AD. This astrocyte module was also linked to neuron-related processes including “brain-derived neurotrophic factor (BDNF) signaling pathway”, “negative regulation of neuron projection development”, and “negative regulation of axonogenesis”. These neurogenesis-specific terms emphasized negative regulation, suggesting that neurogenesis processes are more active in healthy astrocytes. BDNF is a growth factor critical to CNS development, however it also promotes the activation of astrocytes and microglia in neuroinflammation^36^. Within this module we identified enrichment for biological pathways related to copper ions, including “response to copper ion”. Astrocytes regulate the homeostasis of copper in the brain^37^, and copper has been shown to be present at higher levels elevated in AD senile plaques^38^. Copper levels beyond the handling capacity of astrocytes may initially result in a cascade of protective events to reduce labile Cu neurotoxicity, thus activating astroglia, which has been termed the “aberrant copper homeostasis hypothesis”^39^.

The third astrocyte module emphasized apoptosis, or programmed cell death, in response to stress signals, with enriched gene programs including “negative regulation of apoptotic process”. Recent studies show that astrocyte apoptosis may contribute to pathogenesis of many acute and chronic neurodegenerative disorders, such as cerebral ischemia, AD and Parkinson’s disease^40^, and astrocyte apoptosis has also been correlated with “senile plaques” in AD^41^.

Taken together, our disease-predictive model highlighted three separate modules in astrocytes, related to the maintenance of the integrity of cellular barriers such as the blood-brain barrier, to a cellular response to copper, and to apoptosis.

In oligodendrocytes, the leading AD-predictive module was related to neuron regulation, with enriched gene programs including “negative regulation of neuron projection development”, “negative regulation of axonogenesis”, “positive regulation of neuron death”, “dendritic spine maintenance”, and “myelination”. Oligodendrocytes are known to interact closely with axons as they form the myelin sheath^42^, and our results highlight the negative regulation of these helper processes in the disease state, coupled with the regulation of neuronal death, which is a key marker of AD pathogenesis^43^. The second module was related to actin cytoskeleton organization, with enriched gene programs including “negative regulation of stress fiber assembly” and “negative regulation of cytoskeleton organization”. Myelin loss was one of the earliest reported observations in the post-mortem AD brain^44^. Cytoskeleton reorganization and the regulation of actin are key to the myelination process^45^. Our results emphasized that the negative regulation of these processes is predictive of the AD condition. The third module was associated with a cellular response to stress, including “cellular response to cytokine stimulus”, “regulation of cell death”, and “regulation of cellular response to stress”. Amyloid-beta has been shown to drive oligodendrocyte death^46^. Our results identified three modules that reflected the dysregulation of oligodendrocytes in processes relating to neuron maintenance and myelin production, and their death induced by external stressors.

In OPCs, the leading AD-predictive module was related to neuron development and oligodendrocyte differentiation. Enriched gene programs related to neuron development included negative regulation of neuron projection development, axonogenesis, and neurogenesis. The gene program relating to differentiation was “oligodendrocyte specification and differentiation leading to myelin components”. The second gene module was related to cell migration and adhesion. Gene sets included neuron migration and regulation of cell-matrix adhesion. OPCs divide in the adult brain to form new oligodendrocytes for myelin repair in response to myelin damage^47^, and it has recently been shown that OPCs disruption occurs early in a mouse model AD pathogenesis^48^. Our results underlined the involvement of the OPC to oligodendrocyte differentiation process, as well as implicating the interaction between OPCs and neurons.

In excitatory neurons, the leading AD-predictive module was linked to amyloid-beta, GABA neurotransmitter signaling, and presentation of extracellular antigens via MHC. Accumulation of amyloid-beta plaques in neurons is one of the hallmarks of AD, regulated by APOE transport from astrocytes^49^. The second module was related to synaptic growth and transmission, with enriched gene programs including BDNF signaling, positive regulation of synaptic transmission, and cell morphogenesis involved in neuron differentiation. The third module was related to nervous system development and protein phosphorylation. The fourth gene module was related to apoptosis/autophagy driven by interleukins/cytokine signals. Enriched gene programs include “apoptotic process”, “regulation of cell death”, VEGF-A signaling pathway, and multiple terms related to interleukin signaling, specifically IL-12 and IL-18. Neuronal death by apoptosis is one of the most prominent hallmarks of AD^50^, and VEGF-A exposure is linked to neuronal apoptosis in response to injury^51^. Studies have shown pro-inflammatory IL-18 to co-localize with amyloid-beta plaque in AD brains, and increase amyloid-beta production in neuron-like cells^52^. Gene program enrichment results for excitatory neuron modules implicated various forms of neurotransmission and neuron development, along with processes related to neuron death.

In inhibitory neurons, the top AD-predictive module displayed similarities with the top module for excitatory neurons, suggesting a common primary response between the two cell-types. This module was related to GABA signaling and exogenous peptide presentation via MHC class II. The second module related to peptide biosynthesis and autophagy. The third module was enriched in gene programs related to glycolysis and synaptic transmission, as well as the ferroptosis pathway, a form of iron-dependent cell death^53^. All three modules were enriched in gene programs relating to glycolysis and mitochondria. These included “mitochondrial dysfunction-associated senescence” and “regulation of protein insertion into mitochondrial membrane involved in apoptotic signaling”, and “mitochondrion organization”. These results suggested that there is overlap in the response of inhibitory and excitatory neurons to AD. However, we also identified different modules relating to peptide biosynthesis, autophagy, and glycolysis. Additionally, we investigated whether our modules were associated with the canonical inhibitory neuron subpopulation markers *SST*, *VIP*, *KIT*, and *PVALB*^54^ (Supplementary Fig. 1). Using Spearman’s correlation coefficient, we found that each inhibitory subpopulation marker had an above-chance association with at least one of our four gene expression modules (Supplementary Table 1), suggesting distinct relationships between the markers and modules. The marker association was relatively weaker in the top module of inhibitory neuron cells, supporting the idea that this module captures a broad response that affects both excitatory and inhibitory neurons.

**Fig. 3:**
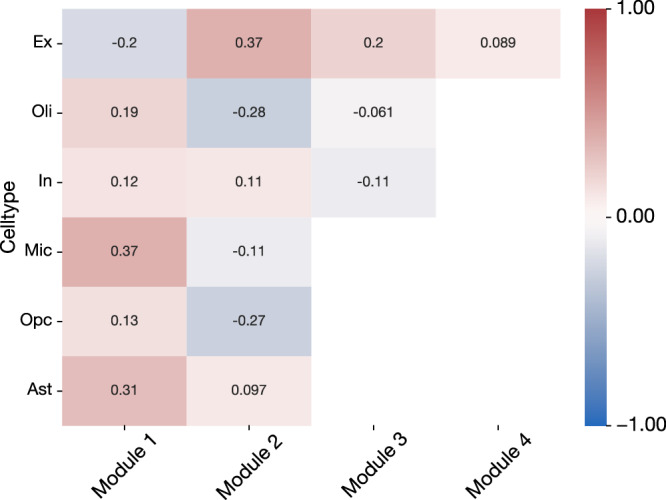
AD-predictive modules show low correspondence with DEGs. We quantified the association between the gene weights in our multivariate AD-predictive modules and univariate differential expression analysis. We calculated the Spearman correlation between each cell-type-specific module and the per-gene expression fold-change (Wilcoxon rank-sum test) for that same cell-type. Differential expression analysis produces one gene ranking per cell-type (vertical axis), whereas our approach results in multiple complementary gene rankings per cell-type (horizontal axis). We found that generally there was a low agreement between the two methods, underscoring the fundamental differences between univariate and multivariate approaches and their potential to identify different signals of scientific interest.

To contrast our proposed method with differential expression analysis, we compared the genes identified by our AD-predictive modules and the genes identified by differential expression analysis. We found that there is generally a low correspondence between the DE results and our modules (Fig. 3), which underscores the novelty and complementarity of our present work. We then compared the top ten genes in each of our cell-type modules (by absolute loading value) with the top ten DEGs. We found that there are no shared genes in any module when compared to the top DEGs ranked by fold change, which indicates that our model is not simply picking up the genes that have the largest magnitude in the expression change. When compared to the top DEGs by significance (ranked by smallest FDR-corrected p-value), we found a subset of genes that overlap, many of which are recognizable as genes with well-known AD associations (Table 1).

**Table 1:** Top module genes overlap with subset of most significant DEGs

	Module 1	Module 2	Module 3	Module 4
Excitatory	*NGFRAP1, BEX1*	*RASGEF1B, LINGO1, SLC26A3*	None	*RAB3A*
Inhibitory	*NGFRAP1, NDUFA4, TMSB4X, PEBP1*	*RASGEF1B, LINGO1, SLC26A3*	None	–
Oligodendrocyte	*SPP1, MID1IP1*	None	None	–
Microglia	*APOE, SPP1*	None	–	–
Astrocyte	*FTH1, TENM2, TMEM241, NRXN1*	None	–	–
OPCs	*OLIG1*	*KCNIP1, NAV2*	–	–

Interpretation of differential expression analysis results typically focuses on the most significantly differentially expressed genes (DEGs) in each cell-type. We tested for overlap between the top ten DEGs in each cell-type (ranked by FDR-corrected p-value) and the top ten genes in our AD-predictive modules (ranked by absolute gene loadings). We find that although only a small number of genes overlap in each cell-type module, many of those that are prioritized by both analysis methods are recognizable as well-studied AD-associated genes.

### Module enrichment reflects relevant AD-associated gene programs

**Fig. 4:**
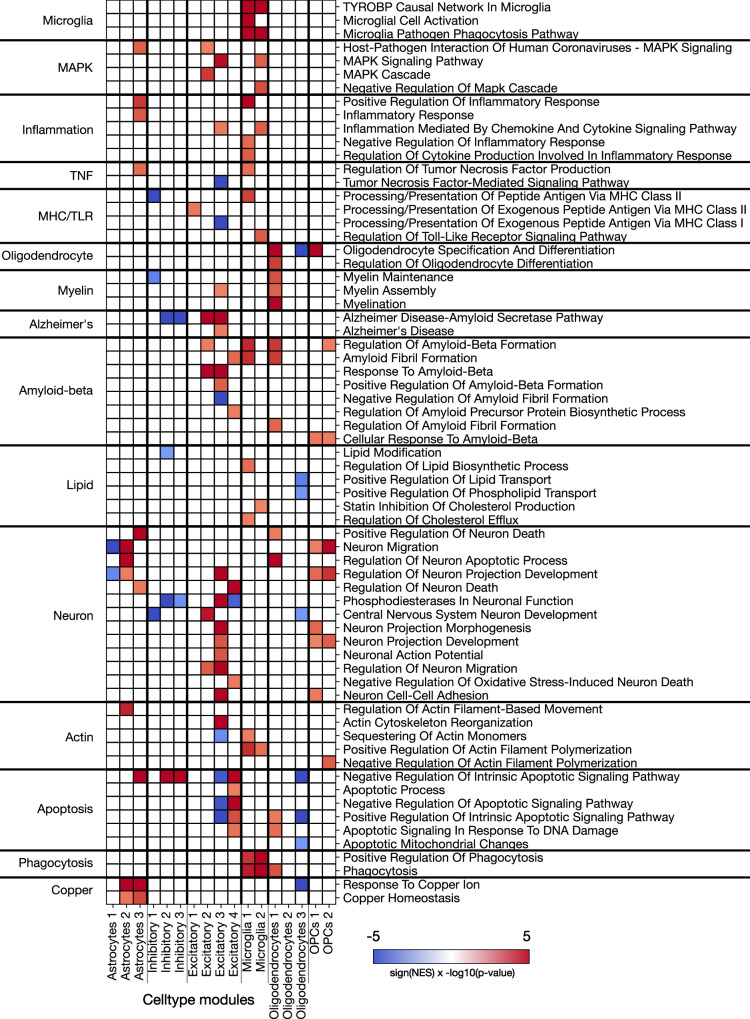
Annotation of AD-predictive gene modules identifies biological processes and pathway. We performed gene set enrichment analysis (GSEA) separately for each cell-type module, ranking the genes by their PLS-DA model weight (cf. Fig. 1). Enriched gene combinations were selected based on significance testing (thresholded at 0.05, FDR-corrected two-tailed p-values). To obtain a synopsis of the mined annotations for each gene module (columns), we searched for keywords relevant to AD and neurodegenerative disorders within the gene set names and charted (heatmap) in which cell-type modules these keywords appear. Specifically, we searched for keywords relating to microglia, the MAPK/ERK pathway, inflammation, tumor necrosis factor (TNF), major histocompatibility class (MHC), toll-like receptors (TLRs), oligodendrocytes, myelin, Alzheimer’s disease, amyloid plaque, lipid metabolism, cholesterol metabolism, neurons, actin, apoptosis, phagocytosis, and copper. We found that over-expressed collections of genes relating to these keywords appeared in biologically plausible cell-type modules. For example, we found that microglia-specific gene programs and those related to phagocytosis appeared almost exclusively in microglia modules. Gene programs related to oligodendrocytes and myelin appeared in the top oligodendrocyte and OPC modules. This comprehensive high-level investigation also revealed gene programs related to neuron death/apoptosis present in astrocyte and OPC modules, and phagocytosis gene programs concentrated in microglia modules and the top oligodendrocyte module. Red indicates a positive normalized enrichment score (NES), blue indicates a negative NES; the color scale indicates the -log10(p-value) of the enrichment. Redundant annotations, due to the hierarchical nature of the ontologies, have been removed for clarity.

To examine the high-level distribution of enriched gene programs across AD-predictive modules from a complementary perspective, we performed a search for specific keywords within the enriched gene program annotations originating from the Gene Ontology (Biological Processes), WikiPathways, and Panther Pathways. In the preceding analysis, we sought to identify overarching categories that summarize the enrichment results in each cell-type module. Here, we instead targeted specific gene programs of interest and explored in which modules they appeared. This approach provided a compelling overview of the processes implicated across all cell-types (Fig. 4). The localization of enriched gene programs to cell-type modules in a biologically plausible fashion provides strong evidence that the learned modules are biologically meaningful.

Cell-type-related: We obtained confirmatory evidence that gene programs linked to the keyword “microglia” in the queried databases were exclusively localized to the microglia-specific modules in our present analyses. Similarly, we found that terms/pathways containing the keyword “oligodendrocyte” were uniquely localized to oligodendrocyte and OPC modules.

Amyloid-beta-related: We observed gene programs related to amyloid-beta across many modules. These fell into two broad categories: amyloid-beta formation (e.g. “regulation of amyloid-beta formation), and the response to amyloid-beta (e.g. “cellular response to amyloid-beta”). The annotations related to amyloid-beta formation that we found in excitatory neuron, microglia, and oligodendrocyte modules, whereas the annotations related to response to amyloid-beta were identified in OPCs and excitatory neurons.

AD-related: Gene programs that have terms explicitly naming Alzheimer’s disease were only found in the modules for inhibitory and excitatory neurons. The localization of the “Alzheimer’s disease” and “Alzheimer’s disease—amyloid secretase” annotations (both from the Gene Ontology) to neurons is expected because the gene annotations for this pathway are almost exclusively those identified in neuron cells.

Immune-related: Because of accumulating evidence^55^ that the cellular immune response plays an important role in AD pathogenesis, we searched for keywords related to immune pathways and signaling across the identified modules. Gene programs containing the term tumor necrosis factor (TNF), which is an immune signaling cytokine, were found in astrocytes, microglia, oligodendrocytes, and excitatory neurons. Programs related to TNF production (such as “regulation of tumor necrosis factor production”) were found exclusively in microglia and astrocytes, whereas those related to a biological response (such as “cellular response to tumor necrosis factor”) were exclusively present in oligodendrocytes and excitatory neurons. Gene programs related to the major histocompatibility complex (MHC) were identified in modules for all cell-types except OPCs. Annotations for MHC class I, which present protein fragments from within the cell, were found in astrocytes, oligodendrocytes, and excitatory neurons; annotations for MHC class II, which serve antigen presentation by displaying protein fragments on the cell surface, were present in microglia and both types of neurons. All gene programs for both MHC class I and II are specific to the presentation of exogenous peptides. It has been found that MHC class II expression is increased in early-stage AD, and this corresponds to an increase in the corresponding protein^56^. Specifically observed in neurons, it has been found that *APOE* acts as an upstream regulator of neuronal MHC class I expression in AD^57^.

Annotations containing the term “immune” emerged predominantly in our microglial modules, with the exception of two gene programs related to neutrophils. These gene programs, “neutrophil mediated immunity” and “neutrophil activation involved in immune response”, appeared in microglia, astrocytes, and excitatory neurons. Neutrophils cross the BBB in AD, and neutrophil granule proteins *CAP37* mRNA has been observed in brain cells including human primary neurons and microglia. *CAP37* is expressed within neurons, and neutrophil elastase and cathepsin G have been detected in microglia, these proteins could be released from neurons or microglia^58^. The observed concordance of neutrophil-related gene sets across multiple cell-types suggests a direction for further investigation to understand their involvement in AD-related processes.

Neuron-related: As would be expected, there were many neuron-related gene programs enriched in excitatory and inhibitory modules. Among these, a subset specifically associated with neuron development also appeared in astrocytes, oligodendrocytes and OPCs.

Apoptosis-related: Enriched gene sets related to apoptosis appeared almost exclusively in excitatory and inhibitory neurons. Both types of neurons contain terms linked to apoptosis in response to DNA damage and the intrinsic apoptotic signaling pathway. This DNA-induced apoptosis of neurons has long been linked to AD^59^.

Phagocytosis-related: Enriched gene sets related to phagocytosis were found primarily in both microglia modules, as well as in the top oligodendrocyte module.

### Cell-type modules enable estimation of disease pseudo-progression

**Fig. 5:**
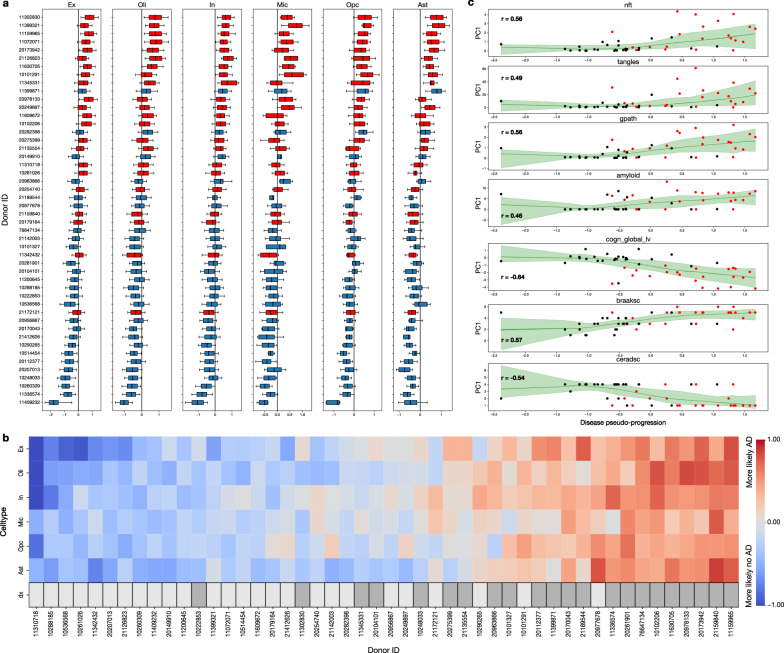
Established clinicopathological markers validate our disease pseudo-progression trajectories. The model-derived AD-prediction estimates for each cell-type indicated that cell-type-specific model’s confidence in the AD vs no AD classification. The strength of gene expression patterns of each cell-type reflected the signatures identified in the predictive modules. **a** Each cell has its own estimated AD prediction confidence, which we then aggregated across all cells of the same cell-type (columns) and from each subject (rows), resulting in a distribution of the predictive confidence. This distribution is due to the variation in prediction confidence for the cells within each subject. Boxes show median and middle quartiles, whiskers show 10/90% range. **b** Under the hypothesis that cells that could be more confidently discriminated between the healthy and AD class were more severely affected by the disease, these effect-strength confidence distributions allowed us to order subjects from least severely affected to most severely affected by AD. The bottom row corresponds to the original binary diagnosis: patient (light) vs. control (dark). **c** We found that this ranking, which we call ‘disease pseudo-progression’, aligned well with both the diagnosis information and with external pathological disease metrics, which were not available to the model during training. We Spearman correlated the pseudo-progression with quantitative post-mortem assessments of neurofibrillary tangles and amyloid plaque, as well as clinical cognition level scores, and found that these were highly correlated. Red (black) dots correspond to persons with (without) AD, and 1/99% bound is shaded.

Taking inspiration from pseudotime methods in the snRNA-seq literature, we next considered cells as different snapshots along a continuous disease trajectory^60^. AD progresses slowly, with pathological changes in the brain beginning years to decades before clinical diagnosis^61^. The 24 persons with AD selected for this study displayed a range of pathology severity, which presented an opportunity to move beyond the binary diagnosis in a way that accounted for this pathology spectrum. Our PLS-DA model was trained to classify cells into two categories—no AD or AD—based on their transcriptomes. The predicted classifications, i.e. the fitted response values, were the result of thresholding the model’s continuous prediction, a value which can be interpreted as the level of confidence the PLS model has in its AD prediction. In the absence of samples at multiple timepoints from the same subject, direct use of this un-thresholded prediction presented an opportunity to quantify disease progression. For each cell-type-specific disease-classification model, we grouped all continuous prediction values by subject (Fig. 5a). We used the out-of-sample predictions accumulated over the five folds of the cross-validation process, ensuring that all estimates were obtained from unseen cells. Because this grouping aggregates predictions across cells originating from a given subject, we obtained an AD diagnosis confidence distribution for each subject. We then ranked the subjects based on the median of these distributions, from most confidently predicted to not have AD to most confidently predicted to have AD (Fig. 5b). Each model was trained on cells from all 48 subjects, so this ranking inherently represents a relative comparison between subjects based on the AD-predictive modules present in each cell-type. The relative strength is determined by the combination of all module’s correspondence with the gene expression patterns observed within a subject’s cells.

We called this ordering of subjects the disease pseudo-progression, as it is an estimate, based only on gene expression, of the severity of each subject’s progression through the stages of AD. To validate whether this pseudo-progression alignes with the biological reality, we compared the resulting subject ordering to widely used clinical and pathological metrics available for each subject in the ROSMAP resource: Braak stage, CERAD score, as well as measured levels of neurofibrillary tangles and amyloid plaque, and assessed global cognition level (Fig. 5c). These quantitative disease progression metrics were at no point available to the PLS-DA model during training, and hence provide the opportunity for an unbiased point of comparison. We found a strong absolute Spearman correlation between our disease pseudo-progression and these established biological indicators of AD progression: 0.57 for Braak stage, 0.54 for CERAD score, 0.64 for global cognition level, 0.56 for neurofibrillary tangles, and 0.46 for amyloid plaque.

Using only single cell gene expression data, we deployed our supervised latent factor model to quantify a subject’s position along a disease pseudo-progression trajectory, from healthy to late-stage AD. The biological validity of this trajectory is supported by its high correlation with postmortem pathological variables and external disease severity metrics.

### Potential inter-cell-type coordination in AD

We then moved beyond cell-type-specific analyses to understand whether there existed some quantifiable coordination between pairs of cell-type modules at the level of the 48 study subjects. Prior to the single-nucleus RNA capture and sequencing, all cells from a given subject originated from a small volume of post-mortem brain tissue. We use this fact as justification to make the assumption that each tissue sample may have captured a local micro-environment present within a subject’s brain at the time of acquisition. The presence of this local micro-environment could therefore plausibly permit the interaction of cell-type modules via inter-cell signaling, or the coordinated response of cells of multiple cell-types to a common stimulus.

To infer such potential modes of coordination between cell-type modules, we first quantified the activity level of each enriched gene program within a module. This activity level was obtained by training a simple PLS-DA disease classifier instance for the top 50 gene programs enriched in each module, resulting in 50 classifiers per module. Each simple classifier only had access to the expression levels of the genes from the gene program of interest as input (see Methods). This effectively restricted the predictive capacity of the model to the information carried by that specific gene program. We aggregated the predictions of this gene-program-specific model across all cells from each subject. This produced a mean disease classification for each subject based on the gene expression of that gene set. Next, we calculated the association magnitude via Pearson’s correlation between the per-subject gene program activity (one activity value per subject) for the top 50 gene programs—as measured by normalized enrichment score (NES)—in one cell-type module with the top 50 gene programs in a module from a different cell-type. We used correlation magnitude as we were only concerned with the strength of the association. We hypothesized that if the activity of biological processes and pathways belonging to one module was highly correlated with those of another module across subjects, this provided evidence that there could be a degree of coordination between these two modules, such as if modules from two different brain cell populations exhibit similar responses to a same external stimulation.

**Fig. 6:**
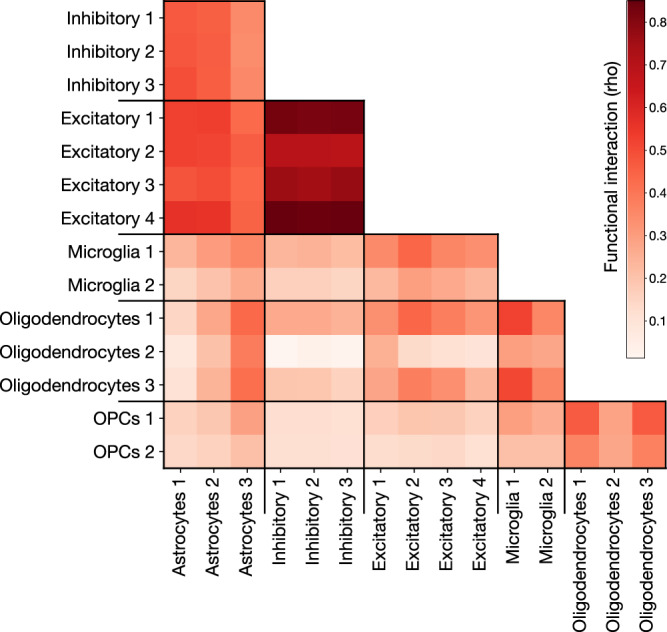
Quantification of the functional interplay between module-specific gene programs. We calculated the association strength between the activity of top enriched gene programs to understand which modules may be functionally interacting with each other across cell-type populations. The “activity” level of the top gene sets in each module for a given subject is the mean across all cells from that subject, where activity level was measured by the ability of the expression level of the subset of genes corresponding to a particular gene set to predict the disease phenotype. Top gene programs were selected based on normalized enrichment score (NES), and the top 50 in each module were used for this analysis. The activity level was aggregated across all cells from each subject. The subject-level mean was taken as representing the extent to which a gene program was active in that subject. We then Pearson correlated these signals between all pairs of cell-type modules, between different cell-types, to estimate which modules showed coordinated changes. We confirmed the expectedly high correlation between all modules of excitatory and inhibitory neurons. We revealed that microglia showed the highest correlation with oligodendrocytes and excitatory neurons, but not inhibitory neurons. Our inter-module analysis demonstrates that coherent activity of different transcriptome modules can be estimated from their gene expression profiles.

Our analysis framework found that the strongest module-module coordination was between all pairs of excitatory and inhibitory modules (Fig. 6), which makes sense as they are cell-types with similar functions that are likely affected by, and respond to, their micro-environment similarly. There was a high level of coordination between all astrocyte modules and both inhibitory and excitatory neuron modules. Disruption of astrocyte-neuron interaction related to synaptic function has been shown to impair memory in a mouse model of AD^62^. Beyond astrocytes, the neuronal cell-type interactions diverged. Excitatory neuron modules exhibited consistently higher coordination with other cell-type modules than those of inhibitory neurons, despite the high coordination between excitatory and inhibitory modules. This suggests that a different subset of gene programs is involved in these interactions, and that excitatory neurons are more involved in oligodendrocyte- and microglia-related dysfunction in AD than inhibitory neurons.

OPC modules showed a low level of coordination with most cell-type modules, only showing elevated levels of potential interaction with oligodendrocyte modules. Similar to neurons, this alignment in responses from similar cell-types reflects their common response to their environment, and suggests a distinct set of gene programs active in both OPCs and oligodendrocytes that are related to AD. Investigating this interaction more closely, we found that the most coordinated gene program pairs across subjects in oligodendrocytes and OPCs were similar: they were related to the negative regulation of neurogenesis, axonogenesis, and neuron projection development.

The top microglia and oligodendrocyte modules showed high levels of coordination with the majority of other cell-type modules, suggesting that they are involved in many different processes in the brain at a cellular level. In general, the level of coordination between modules was similar across all pairs of modules within the same pair of cell-types, except for the second oligodendrocyte module and the top astrocyte module. The second oligodendrocyte module had fewer enriched gene programs than other modules, which limited its ability to extract the subject-level pattern. The top astrocyte module was identified as being related to blood-brain barrier maintenance, and since endothelial cell are not considered in this analysis this interaction would not be expected to appear if it were occurring.

This examination of coordination between AD-predictive cell-type modules, measured through the activity of enriched gene programs, reveals a rich constellation of potential interactions occurring in the brain.

### GWAS risk loci can be localized to a diversity of gene expression modules

In order to further contextualize the AD-predictive modules against established knowledge, we investigated whether AD risk loci identified by genome-wide association studies (GWAS) could be localized to specific cell-types and modules. Given that the heritability of AD is estimated to be around 60–80%^63,64^, it is valuable to understand the specific biological mechanisms that these risk loci are involved in through a single-nucleus, cell-type-module lens. We used the 38 significant risk loci identified by the largest GWAS study of late onset AD to date^3^, as well as the early-onset risk genes *PSEN1* and *PSEN2*, as our target list. We searched for these risk loci in the gene program annotations that were enriched in our AD-predictive modules. Inherited risk variants are present in all cells within a subject, but we hypothesized that they may have different effects in different cell-types and modules. We argue that by intersecting each reported AD risk locus with the gene programs enriched in each module, we can generate a hypothesis about which gene programs present avenues through which an AD-associated variant might influence the disease phenotype.

**Fig. 7:**
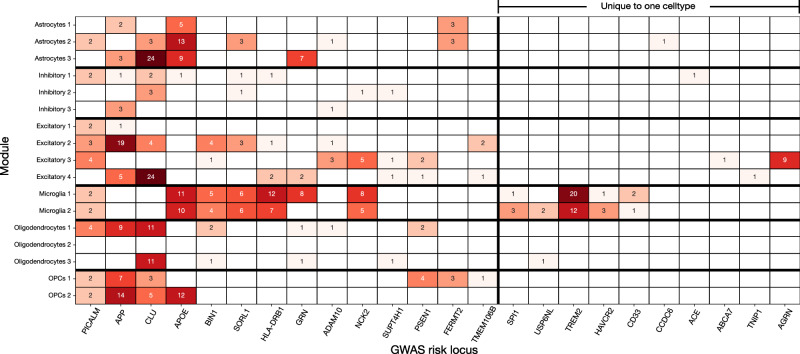
Known GWAS risk loci for AD are implicated in biological pathways specific to gene modules in cell-types. Starting from the enriched gene programs identified in each cell-type by our disease-classification model, we focused on the specific gene programs that implicated known AD risk loci, as reported in a recent GWAS with over one million subjects^3^. We counted the number of times these AD risk loci appeared in the list of genes associated with the enriched gene programs in each cell-type-specific module. This heatmap shows the counts corresponding to each module and risk locus, with the color indicating the magnitude of the locus count. We found that several gene sets containing well-known risk genes appeared in at least one module in most cell-types (*PICALM*, *APP*, *CLU*). There were also many risk genes that were only active in a small subset of cell-types (*NCK2*, *HLA-DRB1*) or a single cell-type (*TREM2*, *CD33*, *AGRN*). Most of the AD-related risk loci that were localized to only a single cell-type were present in microglia or excitatory neuron modules.

We put this hypothesis to the test by searching the AD-predictive modules for the presence of each risk locus and determining in which specific enriched gene programs that risk gene appears. The results of this analysis (Fig. 7) showed that while many of the top AD risk loci were identified in enriched gene programs across the majority of cell-types, a number of the less studied risk loci could be localized to a more focused subset of cell-type modules. Of the 38 loci being considered, 37 were captured in our gene expression data after pre-processing, and 24 appeared in a predictive gene set in at least one cell-type module, including five of the seven never-before reported risk genes (*AGRN*, *TNIP1*, *HAVCR2*, *TMEM106B*, *GRN*). Of the risk loci identified in our AD-predictive modules, one was found in modules in all six cell-types (*PICALM*), two were found in modules in all cell-types except microglia (*APP*, *CLU*), and nine were found in only a single cell-type module. These observations suggest that both cell-type-dependent and -independent effects are important contributors to the disease.

The most numerous risk loci uniquely isolated to a single cell-type were in microglia (*SPI1*, *TREM2*, *HAVCR2*, *CD33*) and excitatory neurons (*ABCA7*, *TNIP1*, *AGRN*). Multiple modules corresponding to these two cell-types contained enriched gene programs that implicated these established AD risk loci. 15 of 24 loci appeared in excitatory neuron modules, and 12 of 24 appeared in microglia modules.

*PICALM* is the only risk gene that was identified across all examined cell-types, where it was almost exclusively enriched in endocytosis-related gene programs. In excitatory neurons, microglia, oligodendrocytes, and OPCs, *PICALM* was enriched in the “regulation of receptor-mediated endocytosis” gene program, while in inhibitory neurons it appeared in “clathrin-dependent endocytosis”, and in astrocytes it appeared in “negative regulation of protein localization to cell periphery”. This coherent signal across cell-types suggests a common role of PICALM, which is known to play a key role in the endocytosis of amyloid-beta^65^. It has been shown that PICALM depletion reduces endocytosis and intracellular APP levels^66^, while increased expression of *PICALM* in iPSC-derived human astrocytes has been shown to reverse *APOE4*-caused endocytic disruptions^67^.

*APOE*, whose e4 variant is the largest risk factor for developing AD^68^, was identified exclusively in the disease-predictive modules corresponding to glial cells (astrocytes, microglia, OPCs), other than a single appearance in a single inhibitory neuron module. *APOE4* is thought to primarily contribute to AD risk by disrupting the homeostatic function of microglia and astrocytes^69^.

In our results, four risk loci were uniquely localized to microglia: *TREM2*, *HAVCR2*, *CD33*, and *SPI1*. *TREM2* and *CD33* are well-known microglia-specific genes. *TREM2* is a highly expressed surface receptor on microglia which binds lipoproteins, in particular *APOE* and *CLU*^25^. It is known to modulate the rate of phagocytosis^24^ and to modulate inflammatory signaling^70^. AD-associated *TREM2* variants have been shown to impair the binding of cell-surface ligands^71^ and alter phagocytic functions^70^. In our results, *TREM2* was enriched in a number of gene programs related to phagocytosis, including “microglia pathogen phagocytosis pathway”, “positive regulation of phagocytosis”, and “positive regulation of kinase activity”, and gene programs related to inflammation, including “negative regulation of inflammatory response”, “regulation of cytokine production involved in inflammatory response”, and “regulation of chemokine production”. *CD33* is a transmembrane protein expressed on immune cells that interacts with the *TREM2* receptor, inhibiting the uptake of amyloid-beta^72^. In our results, it only appeared in the gene programs “neutrophil activation involved in immune response”, “neutrophil degranulation”, and “regulation of tumor necrosis factor production”, found in both microglia disease-predictive modules. Neutrophil granules are involved in neuroinflammation in AD^73^. Microglia are known to protect neurons from infiltrating immune cells by engulfing neutrophil granulocytes^74^. This suggests that microglia may be phagocytosing neutrophil granules in AD, although microglia may also secrete these granule proteins^75^.

*HAVCR2* (also known as TIM-3) is a newly reported AD risk locus which has received little attention in the context of AD. *HAVCR2* can bind to phosphatidylserine on the surface of dying cells, increasing their phagocytosis^76^, and it is also upregulated in activated microglia^77^. In our results it appeared in gene programs related to cytokine signaling: “regulation of tumor necrosis factor production” and “regulation of interleukin-2 production”.

*SPI1* is a transcription factor whose reduced expression in macrophages has been associated with delayed onset of AD^78^. In our results, *SPI1* appeared in the enriched gene programs “regulation of NIK/NF-kappaB signaling” and “RANKL/RANK signaling pathway”. *RANK* is a member of the tumor necrosis factor superfamily and is a main activator of NF-kappaB. Many other prominent AD risk genes have been linked to the NF-kappaB pathway^79^, and our results suggest this connection can be extended to SPI1.

In our results there were three risk loci uniquely enriched in excitatory neuron modules: *ABCA7*, *TNIP1*, and *AGRN*. *ABCA7* is primarily expressed in neurons and microglia in the brain, and is suspected to be involved in cholesterol metabolism and phagocytosis^80^. In our results it is present only in the “phospholipid translocation” gene program, suggesting its role in the lipid clearance. This supports the “altered lipid homeostasis” hypothesis^81^, which proposes that neurotoxic lipids produced in neurons are not adequately cleared when *ABCA7* levels are too low. *TNIP1* is a newly reported AD risk locus, and it is also one of the few known risk loci for the neurodegenerative disease ALS^82^. In our results, it was uniquely enriched in the excitatory neuron gene set “translation”. *AGRN* (Agrin) is also a newly reported AD risk locus. In our results it was primarily found in excitatory neuron gene programs relating to synapses (“synapse organization”, “splicing factor NOVA regulated synaptic proteins”) and “positive regulation of GTPase activity”. Agrin is known to play an important role in excitatory synapse formation/maintenance^83^, and GTPases are also central in controlling this process^84^. Synapse loss is strongly associated with AD and the resulting cognitive decline, and these results suggest that the *AGRN* variant may be involved.

Other AD risk loci of interest in our results included *BIN1*, *TMEM106B*, and *GRN*. *BIN1* appeared in oligodendrocytes, microglia, and excitatory neurons, where across all of these cell-types, it was enriched in the “regulation of amyloid-beta formation” gene program. *TMEM106B* is a newly reported AD risk locus, and it was enriched in gene programs relating to neuron morphogenesis in excitatory neuron and OPC modules, including “dendrite morphogenesis” and “neuron projection morphogenesis”. *GRN* is a newly reported AD risk locus, which in our results was localized to astrocyte, oligodendrocyte, microglia, and excitatory neuron modules, and has been suggested as underlying a shared mechanism for Parkinson’s, AD, and ALS^85^. We also investigated whether the well-known early-onset risk genes *PSEN1* and *PSEN2* were enriched in our predictive modules. Only *PSEN1* appeared, and was localized to excitatory neurons, oligodendrocytes, and OPCs.

By bringing together results from the population level and the cell level, we were able to localize AD risk loci to specific cell-types, AD-predictive modules, and gene programs enriched in those modules. This suggests an under-explored way of contextualizing AD GWAS findings at the resolution of individual nuclei in brain tissue.

## Discussion

Recent snRNA-seq studies have suggested that neuronal and glial cells in the human brain play diverging roles in the onset and progression of AD^5,7,86^. Our present investigation aimed to show that the effects of AD can be further dissected by decomposing the gene expression in each cell-type into distinct coherent modules predictive of AD diagnosis, enabled by a supervised latent factor modeling approach that integrates information from the expression of all genes simultaneously. By examining single-nucleus transcriptomic data through this lens, we demonstrated that dysfunction in the prefrontal cortex in AD can be deconvolved into interpretable cell-type-specific modules. These learned gene expression modules presented opportunities for downstream refinement of the biological conclusions that can be derived from snRNA-seq studies—by direct quantitative examination of gene programs, module-module coordination, and AD GWAS risk loci.

The role of microglia in AD pathogenesis has become increasingly apparent in recent years, spurred by the rapid adoption of snRNA-seq technologies. Our collective findings confirm this direction and extend recent AD studies by detailing evidence as to how microglia may participate in the genesis and progression of AD. In particular, previous transcriptomic studies have found evidence that dysregulated MAPK signaling pathways in microglia contribute to neuroinflammation, impaired phagocytosis, and the subsequent accumulation of toxic proteins, such as amyloid-beta, potentially accelerating the progression of AD^28^. In our analysis, conducted at the granularity of gene expression modules identified within the microglia cell-type, gene program enrichment of the top module pinpointed the activation of microglial cells, phagocytosis, and response to amyloid-beta plaques. The high predictive power of this top module to distinguish AD-affected microglia, relative to modules of other cell populations, underlined the importance of the detected signal. The biological processes and pathways that we identified as being implicated in this gene program may reflect different facets of the amyloid hypothesis of AD. Specifically, amyloid-beta plaque is thought to cause immune activation, and one proposed mode of immune activation is the phagocytosis of this amyloid-beta plaque by microglia^87^. The driving genes in this leading microglia module, which can be thought of as the genes that collectively best explain AD diagnosis in that cell population, replicated several genes well-known to be associated with AD^6^. These included *APOE*, the top risk locus for AD, as well as genes related to the complement system (*C1QA*, *C1QB*, *C1QC*; and *CD14*), a critical regulator of the microglial inflammatory response that acts to modulate Aβ deposition^88^. Gene *CD74* offers a demonstration of the new perspective that comes from isolating coherent patterns of gene expression by searching across all genes simultaneously. While the most salient genes in our two separate microglia modules were distinct, *CD74* was one of the top genes in both modules—playing a role in antigen presentation and a marker of microglia activation^89^. *CD74* has been shown before to be upregulated in microglia in AD^90^. Our results confirm and provide nuance how *CD74* plays distinct roles, in concert with different companion genes, in parallel mechanisms in AD.

Complementing this leading mode of gene expression in microglia, the second most important gene module in this cell-type singled out a specific set of biological cascades, namely the activation of the MAPK/ERK signaling pathway and the activation of toll-like receptors (TLRs). Targeted immunohistochemistry studies have shown that TLRs on microglia can recognize amyloid-beta aggregates, and, upon binding, trigger the MAPK cascade, leading to the production of pro-inflammatory cytokines and exacerbation of neuroinflammation^91,92^. Microglial TLRs have therefore been proposed as a possible target for therapeutic intervention^93^. Our findings consolidate and systematically reframe these previous hints by nominating TLR2, followed by TLR1 and TLR5, as the top predictive receptors identified by means of this microglia module. TLR2 has been found before, via spectroscopy, to be the primary receptor that triggers neuroinflammatory activation in response to amyloid β peptide^94^. Misfolded alpha-synuclein has been shown to trigger the TLR1/2 heterodimer to induce a proinflammatory microglial phenotype in Parkinson’s, hinting at possible overlap between these two neurodegenerative diseases^95^. Another immunocytochemistry study showed that TLR5 activation in microglia modulates their function and contributes to orchestrating immune processes in the brain^96^. Examining commonalities with other cell-type modules, we found that the only other cell-type in which annotation enrichment profiling identified the MAPK cascade are excitatory neurons. The only other cell-type module in which phagocytosis processes were flagged was the top module in oligodendrocytes. As a conjunction of present and previous findings, we have carefully located the involvement of TLR2, TLR1, and TLR5 in the activation of MAPK/ERK signaling pathways in microglia. Our elaboration of previous results of targeted immunological studies single-nucleus expression modules reinforces the potential value of these TLRs in therapeutic strategies for AD.

Expanding our analysis framework to target the coordination between cell-type modules, we provided further context for how our microglia modules may be linked to pathway and biological process annotations of distinct gene modules from other cell-types. Our results suggest that excitatory neurons and oligodendrocytes were the two cell-types with the strongest expected functional liaison with our microglia modules. Because this coordination is based on the aggregated gene expression of disease-predictive gene programs, it draws a more complete picture of AD pathophysiology with coordinated microglia-excitatory-neuron and microglia-oligodendrocyte cell responses. As one tempting explanation, such concerted action could be related to the aforementioned MAPK cascade and phagocytosis, both of which could be triggered as a reaction to external factors present in the cells’ microenvironment. One of the reasons for the expanding focus on microglia is that a majority of AD risk loci discovered through genome-wide association studies were noted to locate in or near genes that are most highly expressed in microglia^97^. Consolidating and extending these previous cues, our analyses showed that the genes *SPI1*, *TREM2*, *HAVCR2*, and *CD33* appear as top disease-predictive features specific to microglia. Bolstering our module-level insights and their functional interaction signatures, all four of these target genes have direct links to phagocytosis and the MAPK cascade. Taken together, combining ontologies of thousands of biological processes and functional interaction analyses, several of our module-resolved findings point to phagocytosis and the MAPK cascade in microglia playing an important role in AD pathogenesis. These identified mechanisms potentially act in concert with dedicated AD-linked gene programs in specific subsets of brain cells including oligodendrocyte and excitatory neuron populations in brain tissue.

Turning attention to the other examined cell-types, we applied the same module-module coordination analysis to chart the coherent activation of AD-predictive gene modules. On a confirmatory note, we find an expectedly strong level of interplay between excitatory and inhibitory neuron modules. Yet, we observed diverging levels of interactions between these two neuronal cell-types and their supporting glial cells. Only astrocytes showed a high correspondence of biological pathways at play in AD with both inhibitory and excitatory neurons. Other glial cell-types (i.e., microglia, oligodendrocytes, OPCs) showed stronger functional engagements with excitatory neurons. This suggests that a different subset of expressed genes may perhaps drive the excitatory-inhibitory neuron coordination as compared to excitatory-neuron-glia synergy. Furthermore, our diverging coordination constellations suggest that excitatory neurons are more involved than inhibitory neurons in dysfunctional processes associated with oligodendrocyte and microglia modules in AD. To our knowledge, this relationship has not been suggested by other single-nucleus AD studies. These results may also help contextualize a recent study that showed, using magnetoencephalography, an association between excitatory circuits and tau depositions, and inhibitory circuits and amyloid-beta depositions^98^. As such, our collective findings suggest that different classes of neurons can be considered as partly independently acting culprits in AD.

In addition to neuronal cell-types, we also identified several patterns of coordination between glial cell-types. Oligodendrocytes were the only cell-type with modules that were found to potentially functionally coordinate with OPCs. Indeed, within these pairs of modules the top gene programs were similar and related to neurogenesis. This suggests that another multi-cell-type response in AD may implicate OPCs shifting from a homeostatic state to aid oligodendrocytes to react to damage inflicted in the course of AD^99^. Our top microglia and oligodendrocyte modules both showed intimate coordination with the majority of other glial cell-type modules. This observation suggests that these modules act in union with many different processes in the brain at a cellular level. Because cell-type–cell-type interaction analysis does not attempt to resolve cause and effect, future rigorously designed experiments could target whether our identified coordinated responses between cell-type modules are due to direct interaction between cells. For example, this could be via intercellular signaling channels, or because of a common response to another factor. The confederated interplay between the derived cell-type modules underscores the complex dynamics between transcriptomic processes that may go well beyond isolated actions of individual cell-types.

Machine learning classification models can inherently produce a measure of the confidence of their predictions. In our case it reflected the strength of the AD-discriminative patterns observed in a given cell. We make use of this capability to infer a subject-level pseudo-progression by ranking the 48 persons in the study by the out-of-sample strength of the AD-associated gene expression patterns learned by the PLS-DA cell-type-specific modules. This approach is grounded in the fact that observed cellular transcriptomes that are more confidently predicted as belonging to a person with AD will be more easily distinguished from non-AD cells due to their more pronounced AD-discriminative expression patterns. We showed that the derived trajectories of disease pseudo-progression reflect similar trends in separate classical pathological semi-quantitative indicators of AD progression, including Braak and CERAD stages. This concordance further demonstrates how supervised transcriptomic models can be used towards generating interpretable insights, by tracing out the different kinds of AD progression. This could help to better stratify individuals in future meta-analyses to better subtype and characterize early, intermediate, and late disease states in AD.

Finally, we sought to revisit AD risk loci established via the so-far best-powered GWAS effort^3^ by means of transcriptomic contextualization. To do so, we zoomed in on candidate cell- and module-resolved gene programs through which these risk genes may propagate tides of disease mechanisms. We were able to establish patterns of risk-locus-associated signaling cascades across the landscape of AD-predictive cell-type modules. Our approach sheds light on which risk loci are robustly involved in common or unique cellular processes. We only observed localization of risk loci within a unique cell-type in excitatory neurons and microglia, suggesting that certain inheritable mechanisms are specific to these two cell-types. In microglia, immune-specific risk loci including *TREM2* and *CD33* were confirmed to be involved in gene programs preferentially in this cell-type. However, the actions of the less studied risk loci *HAVCR2* and *SPI1* were also unique to this cell-type. These genes emerged as relevant in both our AD-predictive microglia modules. We specifically localized *AGRN*, *TNIP1*, and *ABCA7* only to excitatory neuron modules. We identified certain risk genes across most or all cell-type modules, suggesting their possible implication in a broader regime of dysfunction. For example, *PICALM* was identified in modules across all examined brain cell-types. *PICALM* is a clathrin-adaptor protein that is known to play a critical role in clathrin-mediated endocytosis and autophagy, involved in clearance of amyloid-beta plaque^100^, suggesting the possibility of its contributions to a broad response across cell-types. We also identified *APP* and *CLU* in modules in all cell-types other than microglia. Instead, *APOE* was primarily localized to glial cell modules, specifically astrocytes, microglia, and OPCs. These insights attest to the value of using snRNA-seq to see into sub-cell-type module granularity. The value of these findings strengthens the argument for using single-nucleus transcriptomics to assist in contextualizing AD risk loci that have been identified in large-cohort efforts.

## Methods

### Preprocessing of single-nucleus source data

We here analyzed a uniquely rich gene expression data resource: the first single-nucleus RNA sequencing (snRNA-seq) study of AD^5^, which used post-mortem brain tissue from the prefrontal cortex (BA10) of human subjects from the Religious Orders Study and Rush Memory and Aging Project (ROSMAP)^17^. All participants agreed to annual clinical evaluation and organ donation at death. All participants signed an informed consent, an Anatomic Gift Act, and a repository consent to allow for resource sharing. Diagnoses of Alzheimer’s dementia and pathologic AD have been previously reported^101^.

Due to careful experimental design, the transcriptome data exhibits low noise even relative to more recent studies. Additionally, the ROSMAP resource contains a high number of subjects compared to many existing snRNA-seq AD studies. This large subject-level sample size is essential for being able to design and deploy more advanced quantitative analysis frameworks that are able to fully exploit information in subtle expression signals of the major cell populations in the brain. We obtained the filtered single-nucleus transcriptomic dataset from the AD Knowledge Portal (www.synapse.org). This dataset is the result of the pre-processing steps described in the “Quality control for cell inclusion”, “Cell clustering”, and “Cell-type annotation and sub-clustering” sections of the original paper^5^. This dataset provides 70,634 cell transcriptomes from 48 age- and sex-matched subjects (24 males and 24 females, 24 AD cases and 24 unaffected controls) and transcript counts for 17,926 protein-coding genes.

In particular, quality-based filtering of cells and genes was already performed as described in the source study^5^, including the removal of low-quality and outlier cells, and the removal of lowly expressed genes. Only protein coding genes, as opposed to non-coding genes, were kept for downstream analysis. We have built on the cell-type categorization that was provided with a previous study^5^, along with relevant clinical and pathological metadata. These cell-types were identified by clustering the cells using the highly variable genes (based on dispersion and mean), then screening for enrichment of known marker genes (see their Methods section for further details^5^).

In the present study, all further transcriptome preprocessing of the filtered single-nucleus data was performed using the scanpy library^102^. We transformed transcript counts using the default scanpy variance stabilizing transform of log-transforming the scaled counts per cell (scanpy functions pp.normalize_total(data, target_sum=1e4) and pp.log1p(data)). Downstream data analyses of scientific interest were conducted using the scikit-learn python library^103^.

### Extracting functional gene modules indicative of ADRD status: supervised latent factor model

We aimed to identify intrinsic cliques of genes whose cell-type-specific expression robustly covaried with AD diagnosis across the cells in that measured brain tissue population. For that purpose, we brought to bear the class of supervised latent-factor models, which is under-represented in single-cell genomics. The key assumption of latent-factor models is that the patterns in the high-dimensional input data can be captured by a smaller number of underlying hidden factors—that is, weighted linear combinations of the input features (genes) in the context of supervised outcome prediction. This analysis approach is particularly effective if there is appreciable auto-correlation among the input features, which is a well known, but not systematically exploited, property in single-cell RNA-seq genomics^104,105^. Latent-factor models seek to discover and render explicit the quintessential building blocks that jointly compose the gene expression structure by learning these latent factors directly from the transcriptomes themselves. Using a supervised latent-factor model ensures that the discovered factors are traced out in a way such that they uncover principles that can distinguish healthy and AD cells. We thus opted for partial least squares (PLS) as a natural choice of supervised latent-factor model for our present study goals. This model class naturally offers a balance between latent structure discovery capabilities with interpretability in a data-efficient fashion^106,107^.

PLS is a multivariate statistical technique that can be used to deconvolve the many-to-outcome relationship between one potentially large set of (known-to-be correlated) input predictor variables $X$ and a target phenotype (response variable) $y$^106^. In the present study, we utilized PLS for classification purposes, which is also known as PLS discriminant analysis (PLS-DA)^108^. Here, PLS-DA was used to predict the binary AD diagnosis label, separately for each cell-type, based on a set of gene expression features in the subset of cells belonging to one predefined brain cell population (cf. above). All cells sampled from the brains of subjects clinically diagnosed with AD were assigned a positive label (+1), whereas those of the controls were assigned a negative label (−1)^109^. Both matrix $X$ and vector $y$ were subject to column-wise normalization (towards zero mean, unit variance) to facilitate the direct interpretation of the feature weights, given that PLS is a scale-variant approach.

The PLS algorithm identifies and extracts to-be-discovered latent patterns while simultaneously optimizing the emerging latent patterns’ covariance between the biological ambient space $X$ and the target outcome variable $y$. Each latent component is parametrized by a weighted linear combination of all input features^110^. Parameter fitting of a PLS model is rooted in the singular value decomposition of ${X}^{T}Y$. However, if the target $y$ is a single column, the PLS components can be iteratively extracted^111^. This setting involves solving either NIPALS/SIMPLS algorithms or the power method. These solvers are computationally efficient and robust, while allowing the extraction of a specified number of latent components.

Given inputs $X\in {{\mathbb{R}}}^{n\times p}$ and outcome $y\in {{\mathbb{R}}}^{n\times 1}$, where $n$ is the number of observations (cells) and $p$ is the number of measured features (corresponding to gene expression measurements), we find a first major direction of hidden variation in the high-dimensional space $X$ (i.e., a linear combination of its columns) and a scaling of $y$ that has maximum covariance. Let $w\in {{\mathbb{R}}}^{p\times 1}$ and $c{\mathbb{\in }}{\mathbb{R}}$ be the direction vector and scalar corresponding to $X$ and $y$, respectively. Then, by projecting the transcriptome observations onto these emerging latent dimensions, we obtain observation-wise scores $t={Xw}$ and $u={yc}$, such that covariance ${t}^{T}u$ evaluates to a maximum^112^. The loading vector $l={X}^{T}t$ is the first latent component, and represents the contribution of each gene to that particular latent dimension. The information contained in the current latent component emerging from the transcriptome profiles, indexed by (observation-wise) scores and (gene-wise) loadings, is then removed from $X$ and $y$ before extracting the next latent component. By projecting the original transcriptome measurements onto the latent components (cf. below), PLS re-expresses the dataset at a lower dimensionality, while preserving the essential signal of value for outcome classification. Constructing matrices $T$ and $L$ from the collection of individual component scores ${t}_{i}$ and loadings ${l}_{i}$, the original ambient data $X$ can be reconstructed by $X{\approx }{T}{L}^{T}$.

Calculating using the power method, the extraction of latent PLS parameters proceeds as follows:

1. Randomly initialize vector $u$.
2. Iterate: For each successive latent dimension, repeat until $u$ converges:
  - Calculate $w$ as the vector in gene space that maximizes the covariance with the diagnosis label of each cell. This is done by projecting the data columns $X$ (gene expression vectors) onto the emerging estimation of $u$: $w={X}^{T}u$. Intuitively, we thus identify the group of genes whose expression jointly covaries especially with the supervised target outcome (diagnosis). Normalize $w$ to unit length.
  - Calculate the scores $t$, which represent the projection of the rows of $X$ onto $w$: $t={Xw}$. Intuitively, this identifies how strongly cells express the group of genes prioritized by $w$.
  - Calculate the loadings $l={X}^{T}t$.
  - Calculate $c$ as the scalar that maximizes the covariance with $t$. This is done by projecting $t$ onto the outcome vector $y$: $c={y}^{T}u$. Normalize $c$ to unit length.
  - Update $u$ as the scaling of $y$ by $c$: $u={cy}$.
3. Deflate: After a given latent component is found, $X$ and $y$ are deflated by subtracting the outer product of the scores and the loadings from $X$, and the outer product of the scores and the weights from $y$. This is done to remove the portion of variability that is explained by the current latent variable and allow for the extraction of subsequent latent variables.

Finally, from another perspective, the ${m}^{{th}}$ PLS component ${l}_{m}$ is the solution to the optimization objective:

$${\max }_{\alpha }{Cor}{r}^{2}\left(y,X\alpha \right){Var}(X\alpha )$$

subject to |*α*| = 1 and ${\alpha }^{T}{{{{{\boldsymbol{S}}}}}}{l}_{k}=0$, $k=1,\ldots ,m-1$, where ${{{{{\boldsymbol{S}}}}}}$ is the covariance matrix between the columns of X, indexing the degree of gene co-expression strengths across cell observations^110^.

The direct interpretability of loading vectors for each latent factor in PLS classification is crucial for understanding the relative importance and joint influence of gene expression features for AD detection. Gene loadings with large absolute values indicate a strong (marginal) contribution to the overall outcome prediction (AD diagnosis). Loadings with values close to zero suggest little contribution to the pattern encapsulated in a component at hand.

### Cell-type-specific PLS models

Concretely, for the purpose of the present goal to investigate the biological mechanisms at play within each cell-type, we fitted a separate PLS model for all the cells of a given cell-type (scikit-learn cross_decomposition.PLSRegression(scale=True) model). The following procedure was repeated independently for each cell-type prespecified in our dataset. This approach enabled us to partition the overall predictive gene expression signal into coherent latent components that captured intrinsic gene modules specific to each cell-type.

For all observed cell transcriptomes of a given cell-type, we removed genes that were captured in fewer than 1 out of 1000 cells (scanpy function pp.filter_genes()) to adjust the model degrees of freedom by ignoring unexpressed genes. Filtering genes at the level of the cell-type, as opposed to at the level of the entire dataset, ensured that genes that are only expressed in a given cell-type can still be fully considered for downstream analysis. To address any class imbalance in our case vs control setting, the transcriptome observations were subsetted to ensure that the number of cells from each diagnosis class had matched sample sizes. We randomly subsampled cells from the majority class so as to ensure a number of cells was equal in the AD and control groups (scanpy function pp.subsample(), fixed random state initialization for reproducibility). To prevent potential biases arising from the initial ordering of the subjects, at the beginning of the workflow, the entire dataset was shuffled once (scikit-learn function utils.shuffle(), fixed random state for reproducibility). The resulting balanced and shuffled cell-type transcriptomes provided the basis for all downstream analysis steps.

### Model selection via hyperparameter tuning (selecting number of robustly extractable latent components for each cell-type)

The PLS model tuning and ensuing classification performance depend on selecting an appropriate number of latent components, as supported by our data at hand. As each subsequently extracted latent component captures a complementary pattern in the transcriptome profiles, not already explained by a previous PLS component, selecting too few components can lead to poor classification performance and failure to capture informative sets of correlated features. Conversely, selecting an excess of latent components can lead the model to overfit to the training data and capture noise structure, instead of biologically meaningful gene expression signals.

To enable data-informed choices for the number of PLS components and to rigorously assess the model performance for how well we can classify the disease status of cells based on gene expression, we carried out a nested 5-fold cross-validation (CV) scheme. For each cell-type, the transcriptome observations were split into five equal-sized subsets, ensuring an equal number of AD and control cells in each data subset. Four of the subsets were first used for model hyperparameter selection in the inner CV loop, followed by evaluation of the model performance using the final held-out test subset. In the inner CV loop of the nested CV, the (four) combined training subsets were divided into five equal-sized subsets of cells for model tuning. For each combination of training and validation subsets in the inner CV, several PLS-DA models were fitted based on a range of choices for the numbers of components (scikit-learn model_selection.GridSearchCV function with PLSRegression as the estimator and ‘n_components’ parameter set to 1-8). To avoid overfitting, we selected the optimal number of components using the mean area under the receiver operating characteristic (mean AUROC across the inner CV validation subsets). The hyperparameter of the employed PLS instance (number of components) yielding the highest mean AUROC was kept for the subsequent analysis steps. Note that this analysis setup accommodated different numbers of latent components for different brain cell-types.

After determining the optimal number of components for disease detection in each cell-type, a PLS model was estimated on the full set of four training subsets of transcriptome observations and evaluated on the held-out validation subset of the outer CV (scikit-learn model_selection.cross_val_score function). This process was repeated for each outer CV fold. The CV estimates of the expected model performance in new data drawn from the same population distribution was assessed by the mean AUROC across all five outer CV validation subsets. The optimal number of components and ensuing cross-validated AUROC then allowed for a robust and impartial assessment of the model’s performance in distinguishing AD vs. healthy cells based on gene expression profiles.

### Identifying the gene sets most robustly predictive of AD using bootstrap resampling

Next, as a principled assessment of the robustness of each PLS component (loading vector), which indicates the relative roles of the gene transcription features in successful AD detection, we conducted an empirical resampling analysis using a non-parametric bootstrapping scheme. In this analysis, we performed 1000 bootstrap iterations by resampling the cell transcriptomes with replacement. In each bootstrap iteration, the PLS model was fitted to this perturbed version of the original snRNA-seq dataset. The loadings of the corresponding PLS model instance were recorded across bootstrapping iterations—one collection of perturbed PLS model parameter weights, one bootstrap distribution for each gene.

The resulting bootstrap distributions provided an empirical estimate of the model’s loadings for each respective latent component as if we had drawn different transcriptome examples from the original population distribution. This empirically derived non-parametric distribution of effect sizes served as the basis to evaluate the uncertainty of the PLS model’s loading vectors in a disciplined fashion.

### Post-processing of PLS signatures and statistical relevance testing

Based on the derived bootstrap distribution belonging to each gene feature, we determined statistically defensible gene groups, in the context of AD prediction, based on whether the (two-sided) bootstrap loading distribution for each gene, learned simultaneously with all other genes, exhibited robust sets of effects under the 5/95% confidence interval. We silenced genes whose 5/95% confidence intervals of the expression measurements contained zero (i.e., their effect was removed by setting their corresponding loading weight to zero), along with features that had at least one bootstrap loading value of exactly zero.

As a necessary preparatory step, we synchronized the sign directionality of loadings across the different permutation iterations to account for potential mirrored loadings in the bootstrap distribution due to reflection invariance—a form of non-identifiability that is inherent to parts of the PLS model class^113^. To do so, we performed a comparison between the original loading estimates (derived from the original, un-permutated dataset) and the bootstrapped loading vectors in each instance. If the mirrored bootstrap loading vector was more similar to the original loading estimate, as measured by cosine similarity, the loading was aligned by multiplication with -1^113^. This post-hoc adjustment of the obtained PLS parameter estimates aligned the sign of loadings across resampling iterations, ensuring consistency in the polarity of the loadings across the bootstrap iteration-wise PLS instances.

### Comparison of modules with differentially expressed genes

We calculated the Pearson correlation between the gene feature weights (loadings) in each of our cell-type-specific modules (PLS) and the fold-change for the same gene (DEG) in the same cell-type, as obtained by differential expression analysis in the original paper (Mathys et al., 2019, SM4). These differential expression results were obtained as described in the “Differential gene-expression analysis” section of that paper. For each module, we then compared the top ten genes as ranked by absolute loading value with the top ten genes ranked by significance (FDR-corrected p-value) in the DEG results.

### Tracing out the intrinsic directions of variation: PHATE visualization

To allow for a low-dimensional synopsis of the high-dimensional cellular transcriptomes, we performed non-linear dimensionality reduction by means of PHATE^114^. Separately for each cell-type, all corresponding cell transcriptomes were projected into their own independent low-dimensional spaces (scanpy external.tl.phate(k = 15, t=’auto’, a = 100) function with n_pca varying from 5-20 depending on the number of cells). We colored the cells by their PLS score for each latent component (PLSRegression x_scores_), rather than using external variables such as age and sex, as is common in previous snRNA-seq research. The PLS scores quantify the degree of presence of the gene expression pattern for each component. That is, this cell-wise value quantifies how well the gene expression in each cell aligns with gene sets tracking AD status.

### Delineating disease pseudo-progression trajectories

Next, we aimed to understand where individual subjects and their cell transcriptomes lay on the continuous spectrum from health to early-stage to late-stage AD. For that goal, we repurposed the probabilistic predictions provided by the trained PLS classifier as a proxy for how strongly a cell matched the latent patterns that signal AD. Using the cell-type-specific PLS models that were trained to predict binary AD diagnosis based on a cell’s gene expression (cf. above), we grouped all continuous out-of-sample predictions by subject. After per-subject aggregation of the model’s predictions across all cells originating from that subject, we ranked the 48 subjects based on the medians of these prediction distributions.

Taking the median probabilistic diagnosis prediction for each subject produced a (6 cell-types x 48 subjects) matrix. To obtain an ordering of subjects based on their estimated disease progression, we calculated the first principal component of this matrix across subjects and projected each subject onto this principal component space. These rankings order the subjects from those most confidently predicted to be a control to those subjects most confidently predicted to have AD, which we termed “disease pseudo-progression”. The cell-type-specific disease classifiers were trained on cells originating from all subjects, providing a direct and balanced comparison based on the disease-predictive modules present in each cell-type.

To validate the biological relevance of the relative disease progression of subjects derived from their transcriptomic profiles, we calculated the Spearman correlation between the ordered subjects and their available clinical and pathological metrics (Braak stage, CERAD score, amyloid plaque level, global cognition level), as a battery of external metrics of disease progression, commonly used in histological tissue dissection reports in AD^43^.

We employ a classification model’s probabilistic predictive confidence (obtained using a cell’s gene expression) as a proxy for the degree to which AD has affected that cell. This analysis uses the assumption that AD perturbs a cell’s gene expression profile, and that the strength of this perturbation varies as the disease progresses. We believe this is a reasonable assumption, as AD is well established to be a continuously progressing disease, and the progressing phenotype most likely originates from changes at the cellular level. However, it is possible that the model’s predictive confidence correlates with but does not directly correspond to the molecular changes caused by AD. It is also possible that AD only impacts a subset of the cells of a given cell-type obtained from a donor, which may affect the associations based on the total number of cells collected.

### Annotating derived gene modules by known biological signaling pathways: GSEA

We then aimed to gain insights on the biological processes and pathways that are at play in the gene modules (latent components) identified by our cell-type-specific PLS models. For that purpose, it was essential to bring in the biological context provided by annotated gene programs identified by biological experiments. The expression of individual genes is often driven by their membership in complex functional gene regulatory networks.

We performed gene set enrichment analysis (GSEA)^10^ for each component in each cell-type-specific model. We used the following gene set databases, as these resources are the most widely used in existing literature and had the most complete coverage of genes: gene ontology biological processes (2021), WikiPathway human (2021), and Panther pathways (2016). These databases permitted us to respectively explore the enrichment of various biological processes, pathways, and functional categories. We used the python library GSEApy^115^, which itself uses Enrichr^116^. We used the most up-to-date versions of these databases available through Enrichr in October 2022.

For each cell-type and each of its dedicated PLS latent components, we constructed a ranked list of genes based on their corresponding median bootstrap PLS loading. Notably, each gene module’s gene set to be analyzed excluded the zeroed-out gene features (cf. above). We performed gene set enrichment analysis on each ranked list: each candidate gene set was tested for enrichment at either end of the list (gseapy prerank function). This approach was executed using the following parameter choices: minimum gene set size of 5, no plotting, and 1000 permutations for significance testing. We filtered the GSEA results based on a false discovery rate (FDR) threshold of 0.05. The GSEA results for all gene set databases were then combined into a single collection for further analysis and interpretation.

Subsequently, to address the hierarchical nature of the ontologies, we performed an overlap analysis to remove gene sets that were highly similar to other gene sets within the same component. For each pair of gene sets, we calculated the degree of overlap as the ratio of the number of genes shared between both sets to the size of the smaller gene set (overlap threshold of 0.9). If the degree of overlap between two sets exceeded the threshold, the less enriched gene set (by normalized enrichment score) was removed from the results list.

### Gene set keyword search

We performed a string search of the full annotations associated with the GSEA results in order to gain a compact understanding of which types of processes and pathways were enriched in each cell-type component. We searched for annotations that contained the following terms: ‘microglia’, ‘mapk’, ‘inflamm*‘, ‘tumor necrosis’, ‘mhc’, ‘toll-like’, ‘oligod*‘, ‘myelin’, ‘alzheimer’, ‘amyloid’, ‘lipid’, ‘cholesterol’, ‘neuron’, ‘actin’, ‘apopto*‘, ‘phagocyt*‘, ‘copper’. These terms were informed by phenomena commonly associated with AD, as well as our results for the identified themes within the gene modules.

### Characterization of coordination between cell-type modules

To gain a broader perspective over the biological processes and pathways found to be at play in each of the examined brain cell-types, we next inferred a proxy measure for the level of potential coordination between the top enriched gene sets (i.e., biological processes and pathway annotations) discovered in each cell-type. We achieved this goal by first calculating an aggregate activity score, in each subject, for each enriched gene set based on the expression level of its constituent genes. We then identified which of these gene sets had activity scores that associated with our disease pseudo-progression. This approach helped to identify similar biological processes and pathways that plausibly varied together in different cell-types and cell-type modules in a local brain tissue milieu in AD.

To derive a dedicated quantity tracking the activity level of each functional gene set, we first trained a gene-set-focused instance of our PLS model with a single latent component, using only the expression of the genes that were annotated to that enriched gene set as predictors. The target outcome for this gene-set-specific model was binary AD diagnosis. The performance of the classifier indicated how well the purportedly enriched gene set was able to detect diagnosis based on expression levels of its constituent genes. As with the disease pseudo-progression estimate, we used the continuous probabilistic model predictions, here termed the activity score, and aggregated these values across all cells from each subject. Using the mean activity score for each subject, we quantified how predictive the group of genes defined by a given gene set were within that subject, relative to other subjects. This analysis resulted in a gene set activity score for each enriched gene set and each subject.

Finally, we ranked all enriched gene sets based on the Spearman correlation between the disease pseudo-progression (cf. above) scores and the gene set activity scores (both of these scores had one value per subject). Next, according to the derived ranking, we calculated Pearson’s correlation between the activity score vector corresponding to the top 50 gene sets, for all pairs of different cell-types and their corresponding latent components. Then, for each possible pair of cell-types and components, we calculated the median absolute correlation between their top pathway pairs. For example, we identified the top 50 gene sets in module 1 of microglia and module 1 of oligodendrocytes and calculated the correlation between each pairing of gene sets between the two modules, then took the median of these 50^2^ correlation values. This analysis approach was repeated for all possible combinations of transcriptome module pairs.

### GWAS-transcriptome mapping by localizing gene risk loci in cell-type modules

To understand which cell-type-specific gene modules contained enriched gene sets that had as members known AD risk loci identified by GWAS, we interrogated the sets of driving genes in each derived cell-type module. We based this examination on the 38 recently reported loci from the best-powered AD GWAS investigation conducted to date^3^ (Supplementary Table 2). These risk loci are the result of mapping genome-wide variants associated with AD, based on both true and proxy cases, to their most likely associated genes by means of FUMA^117^.

We take this analysis one step further by identifying the most likely cell-types, cell-type modules, and gene sets associated with these AD risk loci. We justify this step by the following argument: for a genetic variant to be associated with a phenotype such as AD, it must also participate in some pathway or process that is itself associated with the phenotype, such as those which are identified in our disease-predictive modules.

For each reported GWAS locus, we counted the number of times it appeared as a member of each AD-predictive gene expression module. To eliminate redundancy due to the hierarchical nature of the ontologies being used, we only kept gene set annotations that were not supersets of other enriched annotations (i.e., only the leaves of the tree of enriched terms). This analysis allowed the identification of the gene sets that were both enriched in our cell-type-specific disease-predictive modules, and contained known inheritable risk loci associated with AD. The ensuing systematic transcriptome-GWAS mappings can be used to help guide further study into which cell-types and via which mechanisms these risk loci may be acting to bring about AD pathophysiology.

### Statistics and reproducibility

Machine learning analyses were performed using 5-fold out-of-sample cross-validation to ensure robustness and generalizability. Results are expressed as mean and standard deviation (s.d.) unless otherwise specified.

### Reporting summary

Further information on research design is available in the Nature Portfolio Reporting Summary linked to this article.

### Supplementary information

Supplementary Information

Reporting Summary

## Data Availability

The snRNA-seq PFC data originated from Mathys, H. et al. Single-cell transcriptomic analysis of Alzheimer’s disease. *Nature* **570**, 332–337 (2019), and are available through Synapse (https://www.synapse.org/#!Synapse:syn18485175) under the doi 10.7303/syn18485175^5^. The data is available under controlled use conditions set by human privacy regulations The numerical source data behind the graphs can be found in 10.5281/zenodo.10962415^118^.

## Code availability

All code to reproduce the analysis and generate the figures in this article are available at: https://github.com/dblabs-mcgill-mila/snRNA-AD_latent_modules^118^. Python package versions are listed in environment.yaml file.
