# Supplementary material for: Supervised latent factor modeling isolates cell-type-specific transcriptomic modules that underlie Alzheimer’s disease progression
Source: Commun Biol. 2024 May 17;7:591. doi: 10.1038/s42003-024-06273-8 (PMC11101463; doi:10.1038/s42003-024-06273-8)
Supplement: Supplementary file 1 — Supplementary Information [file 42003_2024_6273_MOESM1_ESM.pdf]

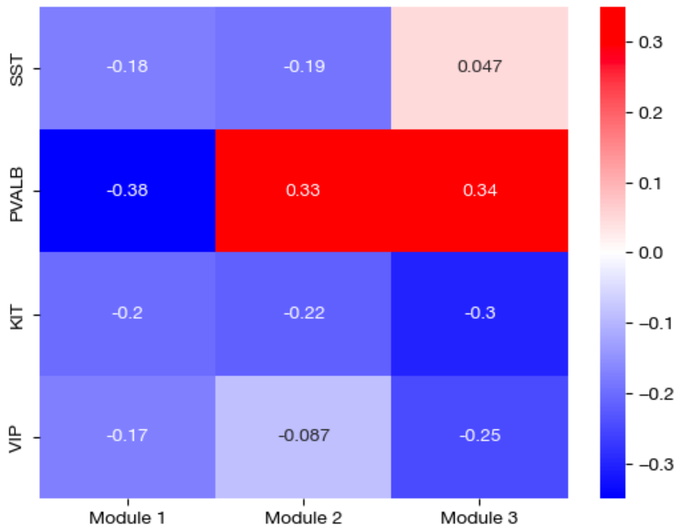

Supplementary Figure 1. Relationship between inhibitory neuron subpopulation markers and modules
We investigated how canonical inhibitory neuron subpopulation markers (SST, VIP, KIT, and PVALB) are related to the AD gene expression modules that we derived for our inhibitory neuron cells. We checked to see if any of our identified inhibitory modules were associated with the expression of these markers at the transcriptome level. We quantified the association by calculating the Spearman correlation coefficient between our module scores per cell (the same quantity that was used to color Figure 2) and the inhibitory neuron marker gene expression level across transcriptome observations. We found that each inhibitory subpopulation marker has an above-chance association in at least one of our four gene expression modules, suggesting distinct relationships between the markers and modules.

	**Module 1**	**Module 2**	**Module 3**
Mean (std dev) absolute correlation across 1000 randomly selected genes	0.20 (0.12)	0.04 (0.04)	0.03 (0.04)
SST	-0.18	-0.19	0.05
PVALB	-0.38	0.33	0.34
KIT	-0.20	-0.22	-0.30
VIP	-0.17	-0.09	-0.25

Supplementary Table 1. Relationship between inhibitory neuron subpopulation markers and modules
To ensure that the associations are biologically meaningful and not the result of random chance variation in our data, we also calculated the mean Spearman correlation between each marker gene expression and 1000 other randomly selected genes, picked from the full transcriptome, for each module.

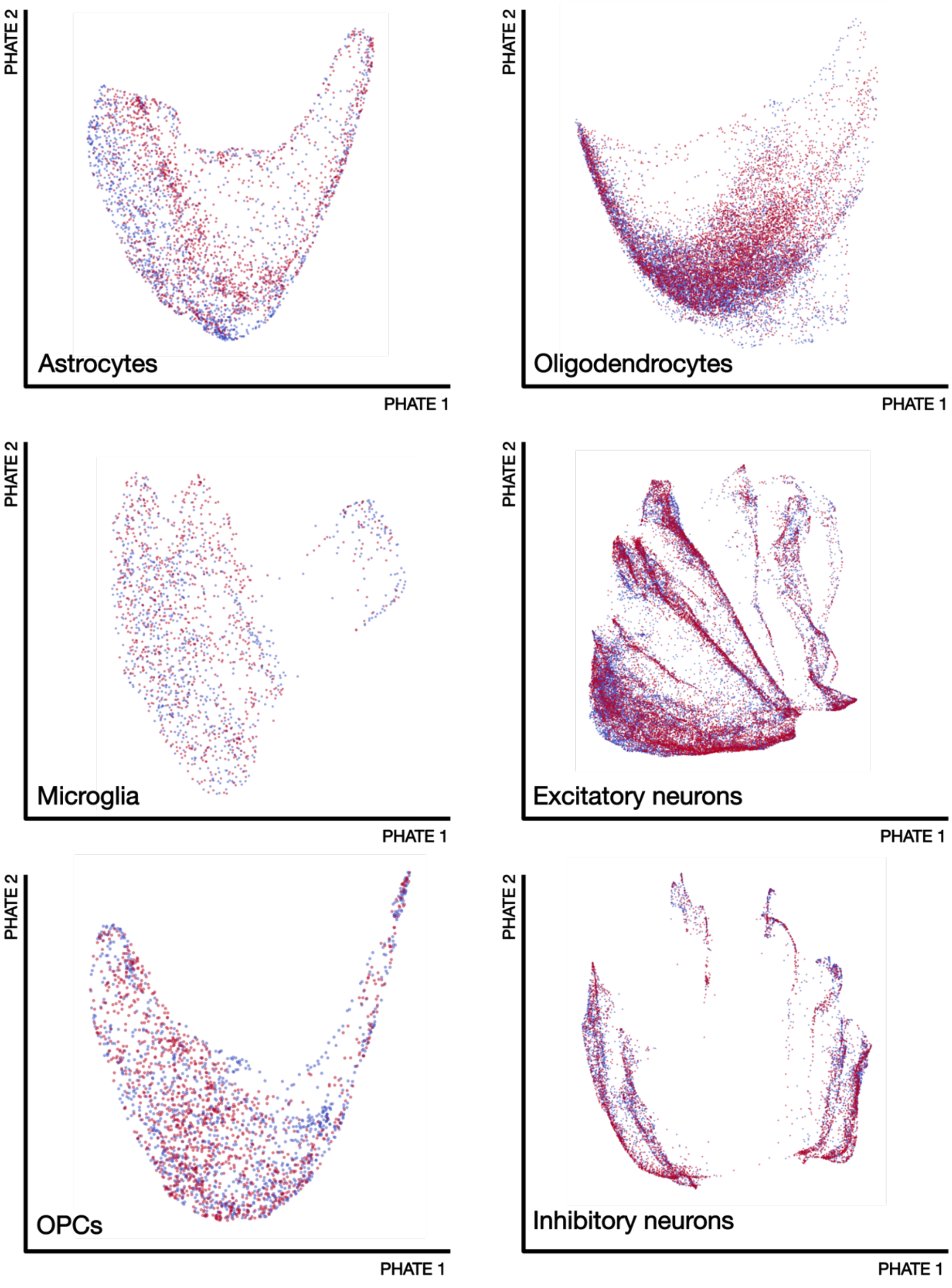

Supplementary Figure 2. PHATE colored by subject diagnosis
As a point of comparison, we show the same PHATE visualizations of the cells as in Figure 2 but colored by the AD diagnosis of the donor (red = AD, blue = no AD).

Gene	A1 frequency	P value
AGRN	0.0041	3.83 × 10−8
CR1	0.82	2.42 × 10−25
NCK2	0.0035	1.33 × 10−8
BIN1	0.41	3.92 × 10−58
INPPD5	0.45	4.65 × 10−9
CLNK	0.79	5.23 × 10−12
TNIP1	0.32	1.37 × 10−9
HAVCR2	0.77	7.91 × 10−10
HLA-DRB1	0.30	2.66 × 10−14
TREM2	0.997	1.26 × 10−25
CD2AP	0.27	1.70 × 10−17
TMEM106B	0.41	2.70 × 10−9
ZCWPW1/NYAP1	0.69	9.41 × 10−16
EPHA1-AS1	0.62	4.69 × 10−11
CLU	0.39	1.57 × 10−22
SHARPIN	0.95	3.14 × 10−9
USP6NL/ECHDC3	0.46	7.68 × 10−15
CCDC6	0.54	3.68 × 10−8
MADD/SPI1	0.54	8.78 × 10−9
MS4A4A	0.62	3.40 × 10−33
PICALM	0.35	1.24 × 10−26
SORL1	0.96	1.33 × 10−13
FERMT2	0.89	6.99 × 10−11
RIN3	0.67	6.63 × 10−17
ADAM10	0.70	6.22 × 10−15
APH1B	0.13	7.00 × 10−12
SCIMP/RABEP1	0.33	3.18 × 10−8
GRN	0.61	1.98 × 10−9
ABI3	0.54	4.90 × 10−10
TSPOAP1-AS1	0.54	7.46 × 10−10
ACE	0.61	1.23 × 10−9

Gene	A1 frequency	P value
ABCA7	0.68	2.81×10^{-15}
APOE	0.84	$<1.0 \times 10^{-300}$
NTN5	0.47	1.72×10^{-8}
CD33	0.37	2.21×10^{-10}
LILRB2	0.49	1.56×10^{-9}
CASS4	0.083	6.73×10^{-16}
APP	0.44	7.66×10^{-10}

Supplementary Table 2. AD GWAS risk genes
AD GWAS risk genes identified by: Wightman, D. P. et al. A genome-wide association study with 1,126,563 individuals identifies new risk loci for Alzheimer's disease. Nat. Genet. 53, 1276–1282 (2021).
