# Supplementary material for: Supervised latent factor modeling isolates cell-type-specific transcriptomic modules that underlie Alzheimer’s disease progression
Source: Commun Biol. 2024 May 17;7:591. doi: 10.1038/s42003-024-06273-8 (PMC11101463; doi:10.1038/s42003-024-06273-8)
Supplement: Supplementary file 2 — Reporting Summary [file 42003_2024_6273_MOESM2_ESM.pdf]

nature portfolio

Corresponding author(s): Danilo Bzdok

Last updated by author(s): Apr 12, 2024

Reporting Summary

Nature Portfolio wishes to improve the reproducibility of the work that we publish. This form provides structure for consistency and transparency in reporting. For further information on Nature Portfolio policies, see our Editorial Policies and the Editorial Policy Checklist.

Statistics

For all statistical analyses, confirm that the following items are present in the figure legend, table legend, main text, or Methods section.

n/a	Confirmed	
☐	☒	The exact sample size (*n*) for each experimental group/condition, given as a discrete number and unit of measurement
☐	☒	A statement on whether measurements were taken from distinct samples or whether the same sample was measured repeatedly
☐	☒	The statistical test(s) used AND whether they are one- or two-sided<br>*Only common tests should be described solely by name; describe more complex techniques in the Methods section.*
☐	☒	A description of all covariates tested
☐	☒	A description of any assumptions or corrections, such as tests of normality and adjustment for multiple comparisons
☐	☒	A full description of the statistical parameters including central tendency (e.g. means) or other basic estimates (e.g. regression coefficient) AND variation (e.g. standard deviation) or associated estimates of uncertainty (e.g. confidence intervals)
☐	☒	For null hypothesis testing, the test statistic (e.g. *F*, *t*, *r*) with confidence intervals, effect sizes, degrees of freedom and *P* value noted<br>*Give P values as exact values whenever suitable.*
☒	☐	For Bayesian analysis, information on the choice of priors and Markov chain Monte Carlo settings
☒	☐	For hierarchical and complex designs, identification of the appropriate level for tests and full reporting of outcomes
☐	☒	Estimates of effect sizes (e.g. Cohen's *d*, Pearson's *r*), indicating how they were calculated

Our web collection on statistics for biologists contains articles on many of the points above.

Software and code

Policy information about availability of computer code

Data collection	No software was used.
Data analysis	python 3.9, scanpy 1.8, scipy 1.8, scikit-learn 1.0, gseapy 0.10.8

For manuscripts utilizing custom algorithms or software that are central to the research but not yet described in published literature, software must be made available to editors and reviewers. We strongly encourage code deposition in a community repository (e.g. GitHub). See the Nature Portfolio guidelines for submitting code & software for further information.

Data

Policy information about availability of data

All manuscripts must include a data availability statement. This statement should provide the following information, where applicable:

- Accession codes, unique identifiers, or web links for publicly available datasets
- A description of any restrictions on data availability
- For clinical datasets or third party data, please ensure that the statement adheres to our policy

The single-nucleus RNA-Sequencing data is available at Synapse (https://www.synapse.org/#!Synapse:syn18485175). The DOI for this dataset is: 10.7303/syn18485175. The data is available under controlled use conditions set by human privacy regulations.

Human research participants

Policy information about studies involving human research participants and Sex and Gender in Research.

Reporting on sex and gender	Individuals were balanced between sexes (24 each), and findings apply to both sexes. Gender was not collected.
Population characteristics	Individuals were balanced between sexes (12 each), and matched for age (medians 86.7 (AD-pathology) and 87.1 (no-pathology)) and years of education (medians 19.5 (AD-pathology) and 18 (no-pathology)).
Recruitment	24 control individuals were selected with no or very little pathology (no-pathology) and 24 age-matched individuals with a spectrum of mild to severe β-amyloid and other pathologies (AD-pathology). The no-pathology group included some individuals with clinical cognitive impairment, as study participants were selected solely based on pathology, with all other variables allowed to freely associate.
Ethics oversight	The Religious Orders Study and Rush Memory and Aging Project were approved by an Institutional Review Board (IRB) of Rush University Medical Center.

Note that full information on the approval of the study protocol must also be provided in the manuscript.

Field-specific reporting

Please select the one below that is the best fit for your research. If you are not sure, read the appropriate sections before making your selection.

☒ Life sciences ☐ Behavioural & social sciences ☐ Ecological, evolutionary & environmental sciences

For a reference copy of the document with all sections, see nature.com/documents/nr-reporting-summary-flat.pdf

Life sciences study design

All studies must disclose on these points even when the disclosure is negative.

Sample size	The dataset contained data from n=48 donors.
Data exclusions	Low quality snRNA-seq libraries were excluded and the exclusion criteria are described in the manuscript as follows. Cells with a high ratio of Mitochondrial (MT) relative to endogenous RNAs had low starting amounts of RNA, which might indicate that source cells were dead or stressed, resulting RNA degradation. Outlier cells in these quality metrics were found to cluster together in the tSNE 2D space . Based on these observations and subsequent scatter plot analyses, cells with less than 200 detected genes, and cells with abnormally high ratio of counts mapping to MT genes, relative to the total number of detected genes were removed. Specifically, given a highly skewed empirical distribution of the MT ratio values (i.e., having an elbow shape clearly separating high and low scores) outlier cells were classified in two groups using the k-means clustering algorithm (k=2) on the MT ratio, and subsequently removed. Identified cell-types that did not appear in all subjects were discarded.
Replication	Out-of-sample cross-validation was used to ensure reproducibility of computational analyses.
Randomization	Data was randomly shuffled for analysis.
Blinding	No blinding was performed.

Reporting for specific materials, systems and methods

We require information from authors about some types of materials, experimental systems and methods used in many studies. Here, indicate whether each material, system or method listed is relevant to your study. If you are not sure if a list item applies to your research, read the appropriate section before selecting a response.

Materials & experimental systems

n/a	Involved in the study
☒	☐ Antibodies
☒	☐ Eukaryotic cell lines
☒	☐ Palaeontology and archaeology
☒	☐ Animals and other organisms
☒	☐ Clinical data
☒	☐ Dual use research of concern

Methods

n/a	Involved in the study
☒	☐ ChIP-seq
☒	☐ Flow cytometry
☒	☐ MRI-based neuroimaging
